# Decay of Skin-Specific Gene Modules in Pangolins

**DOI:** 10.1007/s00239-023-10118-z

**Published:** 2023-05-30

**Authors:** Bernardo Pinto, Raul Valente, Filipe Caramelo, Raquel Ruivo, L. Filipe C. Castro

**Affiliations:** 1 CIMAR/CIIMAR - Interdisciplinary Centre of Marine and Environmental Research, University of Porto, Avenida General Norton de Matos, S/N, 4450-208 Matosinhos, Portugal; 2 Department of Biology, Faculty of Sciences, University of Porto (U. Porto), Rua Do Campo Alegre S/N, 4169-007 Porto, Portugal

**Keywords:** Gene loss, Skin, Sebum, Sweat glands, Immunity

## Abstract

The mammalian skin exhibits a rich spectrum of evolutionary adaptations. The pilosebaceous unit, composed of the hair shaft, follicle, and the sebaceous gland, is the most striking synapomorphy. The evolutionary diversification of mammals across different ecological niches was paralleled by the appearance of an ample variety of skin modifications. Pangolins, order Pholidota, exhibit keratin-derived scales, one of the most iconic skin appendages. This formidable armor is intended to serve as a deterrent against predators. Surprisingly, while pangolins have hair on their abdomens, the occurrence of sebaceous and sweat glands is contentious. Here, we explore various molecular modules of skin physiology in four pangolin genomes, including that of sebum production. We show that genes driving wax monoester formation, *Awat1/2*, show patterns of inactivation in the stem pangolin branch, while the triacylglycerol synthesis gene *Dgat2l6* seems independently eroded in the African and Asian clades. In contrast, *Elovl3* implicated in the formation of specific neutral lipids required for skin barrier function is intact and expressed in the pangolin skin. An extended comparative analysis shows that genes involved in skin pathogen defense and structural integrity of keratinocyte layers also show inactivating mutations: associated with both ancestral and independent pseudogenization events. Finally, we deduce that the suggested absence of sweat glands is not paralleled by the inactivation of the ATP-binding cassette transporter *Abcc11*, as previously described in Cetacea. Our findings reveal the sophisticated and complex history of gene retention and loss as key mechanisms in the evolution of the highly modified mammalian skin phenotypes.

**Supplementary Information:**

The online version contains supplementary material available at 10.1007/s00239-023-10118-z.

## Introduction

**Fig. 1:**
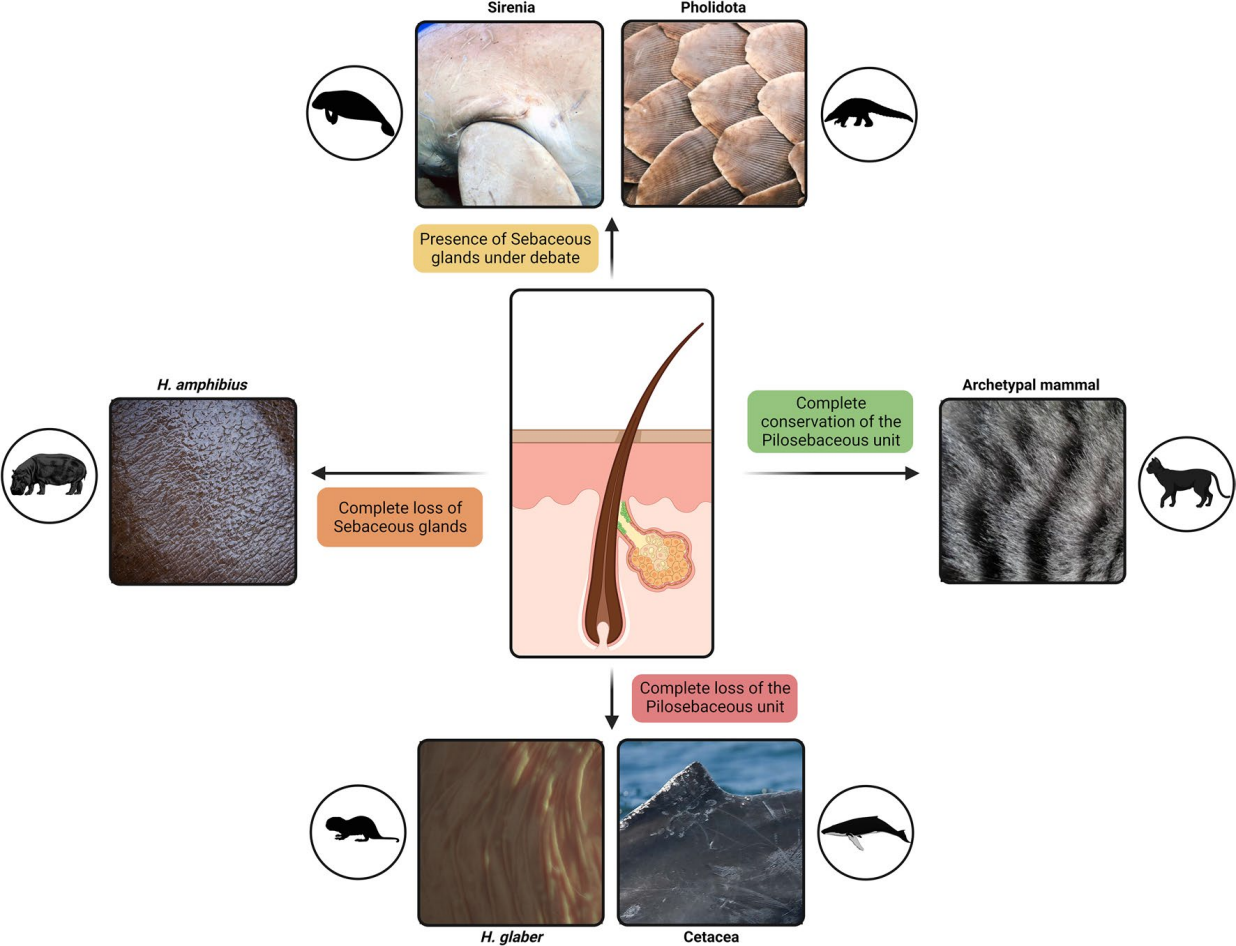
Schematic representation of the mammalian skin diversity, regarding the pilosebaceous gland. Cetaceans and the naked mole rat completely lack the pilosebaceous unit; although sebaceous glands were also lost in hippopotamuses, sparse hair is present. In Sirenia and Pholidota, the presence and degree of functionality of this gland is disputed. Other mammalian species conserve the typical structure of the pilosebaceous gland. Created with BioRender.com

The evolution of mammals entailed some tantalizing lifestyle variations. Ecological transitions such as subterranean burrowing, powered flight, or obligate aquatic regimes, elaborated from prominent eco-physiological adaptations, notably in the skin (Themudo et al. 2020; Wu et al. 2022; Christmas et al. 2023). Some of these skin-phenotypic shifts were quite radical, as illustrated by the complete absence of glands and pelage in Cetacea skin (Fig. 1). The molecular foundations underscoring the skin phenotype of Cetacea is contingent on gene repertoire variations (Nery et al. 2014; Springer and Gatesy 2018; Lopes-Marques et al. 2019a, b; Springer et al. 2021; Themudo et al. 2020; Kowalczyk et al. 2022; Holthaus et al. 2021; Fuchs et al. 2022), which translate into a thick and smooth skin, to counterbalance the mechanical and thermal stress associated with an obligatory aquatic lifestyle (Spearman 1972; Reeb et al. 2007). In other mammalian lineages, the morphological co-occurrence of hair and associated glands (i.e., the pilosebaceous unit) has been more challenging to ascertain. In manatees (*Trichechus manatus latirostris*), for instance, complete sebaceous gland regression is still disputed, while in the semi-aquatic hippopotamus (*Hippopotamus amphibius*) hair is sparsely present yet, sebaceous and sweat glands were so far undetected (Fig. 1) (Sokolov 1982; Graham 2005; Springer et al. 2021). In agreement, the collection of key molecular modules participating in sebum production in aquatic or semi-aquatic mammals was shown to mirror such mosaic of skin morphologies: being fully absent in cetaceans but partially eroded in manatees and hippopotamuses (Lopes-Marques et al. 2019a, b; Themudo et al. 2020; Springer et al. 2021). Extreme skin modifications are not restricted to aquatic species and include the naked mole rat and pangolins (Menon et al. 2019; Li et al. 2020; Savina et al. 2022). Species from the order Pholidota display a formidable keratin-scale armor probably serving as a key deterrent against predators and infections (Fig. 1) (Meyer et al. 2013; Choo et al. 2016; Li et al. 2020). Interestingly, while pangolins have scattered hair on their abdomens, the presence of exocrine glands is contentious, since sweat and sebaceous glands were not detected in their dermis (Liumsiricharoen et al. 2008; Li et al. 2020). Consistently, the reported gene sequence decay of the melanocortin 5 receptor (*Mc5r*) in pangolins (Springer and Gatesy 2018; Liu et al. 2022), a gene with abundant expression in exocrine glands and centrally involved in sebogenesis (Eisinger et al. 2011; Xu et al. 2020; Shintani et al. 2021), is suggestive of a radical shift in skin exocrine function. Overall, the comparative molecular architecture governing skin physiology in particular that associated with the glandular exocrine and eccrine compartment in Pholidota is largely unknown. Here, we provide an exhaustive comparative genomic analysis of the molecular modules governing mammalian skin homeostasis in four species of pangolins. Our findings provide an insight into the richness of evolutionary routes and processes responsible for the skin physiology of extant mammalian lineages.

## Methods

### Genome Resources

The genome regions of the target genes were retrieved from the NCBI (National Center for Biotechnology Information) database. Two of the studied species have high-quality genome annotations (*M. javanica* (GCA_014570535.1) and *M. pentadactyla* (GCA_014570555.1)) (Table 1). Thus, target genes and corresponding genomic regions were retrieved using gene symbols and manually verified. We additionally used two recently released genome assemblies (non-annotated) from *M. javanica* (GCA_024605085.1) and *M. pentadactyla* (GCA_024244205.1) (Wang et al. 2022; Yan et al. 2023). For non-annotated genomes (Table 1), we used BLAST (Basic Local Alignment Search Tool), using as a query the target and two flanking genes from species with annotated genomes, to extract the full scaffold sequence. Gene selection involved an exhaustive literature review to define key genetic pathways involved in skin physiology (e.g., Themudo et al. 2020). This initial screening was complemented with the investigation of the skin “*enriched genes*” list from the Human Protein Atlas resource (Uhlén et al. 2015). Additionally, we used *String* to explore protein–protein networks of skin-specific gene families (Szklarczyk et al. 2021).

**Table 1:** *PseudoIndex* values (0 to 5, with 5 indicating probable mutations leading to pseudogenization; X denotes not found in Pseudo*Checker* analysis) of the studied skin-related genes in pangolin genomes

	*Manis javanica*				*Manis pentadactyla*				*Manis crassicaudata*		*Phataginus tricuspis*	
	GCA_014570535.1 N50 contig: 83,964		GCA_024605085.1 N50 contig: 15,813,259		GCA_014570555.1 N50 contig: 151,937		GCA_024244205.1 N50 contig: 13,973,343		GCA_016801295.1 N50 contig: 7,447		GCA_004765945.1 N50 contig: 23,755	
AADACL3	NW_023436200.1	5	JAMQTK010000009.1	5	NW_023456429.1	5	JAMXTM010005659.1	5	QZMM01052942.1	X	SOZM010018664.1	5
AWAT1	NW_023436150.1	5	JAMQTK010000059.1	5	NW_023454910.1	5	JAMXTM010000791.1	5	QZMM01191925.1	X	SOZM010039185.1	5
AWAT2	NW_023436233.1	5	JAMQTK010000059.1	5	NW_023457172.1	5	JAMXTM010005993.1	5	QZMM01012810.1	5	SOZM010024681.1	5
DGAT2L6	NW_023436233.1	5	JAMQTK010000059.1	5	NW_023454910.1	5	JAMXTM010000791.1	5	QZMM01083316.1 QZMM01012022.1 QZMM01201031.1	5	SOZM010017370.1 SOZM010058764.1	5
EDA2R	NW_023436016.1	5	JAMQTK010000059.1	5	NW_023458050.1	3	JAMXTM010005993.1	5	QZMM01332753.1 QZMM01003526.1 QZMM01124215.1 QZMM01281266.1	X	SOZM010132249.1 SOZM010048560.1 SOZM010091528.1 SOZM010060741.1	5
FABP9	NW_023436188.1	5	JAMQTK010000079.1	5	NW_023455931.1	5	JAMXTM010000891.1	5	QZMM01291322.1	5	SOZM010004921.1	5
GSDMA	NW_023436081.1	5	JAMQTK010000026.1	5	NW_023456908.1	0	JAMXTM010005937.1	5	QZMM01347780.1	5	SOZM010000065.1	0
GSDMB	NW_023436081.1	5	JAMQTK010000026.1	5	NW_023456908.1	5	JAMXTM010005937.1	5	QZMM01024159.1	5	SOZM010000065.1	5
GSDMC	NW_023436052.1	5	JAMQTK010001945.1	5	NW_023458035.1	5	JAMXTM010000300.1	5	QZMM01157587.1	X	SOZM010244792.1	X
GSDMD	NW_023436087.1	0	JAMQTK010002352.1	5	NW_023454130.1	0	JAMXTM010000134.1	5	QZMM01228481.1	3	SOZM010008554.1	5
MOGAT3	NW_023436195.1	5	JAMQTK010000019.1	5	NW_023454636.1	4	JAMXTM010005948.1	4	QZMM01311364.1	5	SOZM010007253.1	5
SLURP1	NW_023436087.1	X	JAMQTK010002352.1	X	NW_023454130.1	X	JAMXTM010000134.1 JAMXTM010005859.1	X	QZMM01029954.1	5	SOZM010015272.1	3
TCHHL1	NW_023436233.1	X	JAMQTK010002013.1	X	NW_023454669.1	X	JAMXTM010000746.1	X	QZMM01320808.1	X	SOZM010020578.1 SOZM010004078.1	X

Genomic location *per* gene (Accession number) in each genome is indicated

### Sequence Alignment Analysis

Each gene sequence was aligned against a coding and curated reference sequence for each gene (*Homo sapiens*) using Pseudo*Checker* (Alves et al. 2020), a program that uses the MACSE alignment software (Ranwez et al. 2018). Pseudo*Checker* attributes a value ranging from 0 to 5, the *PseudoIndex*, with respect to the status of the genomic sequence in comparison to the reference: a value of 0 representing a fully functional protein coding sequence and a value of 5 suggesting pseudogenization mutations (Table 1). For the genomic regions scoring 3 or higher on *PseudoIndex*, a second alignment was performed, against the same reference, using Geneious Prime 2021.2.2 (https://www.geneious.com), for manual curation and evaluation.

### Mutational Validation via SRAs

Identified mutations were validated using Sequence Read Archives (SRAs) (Online Resource 1). Two independent projects were used *per* species when possible and aligned with our genomic sequence using Geneious Prime 2021.2.2. Exceptions include *P. tricuspis*, for which only one SRA is available and *M. crassicaudata* for which no SRA project exists. Cross-species conserved non-synonymous mutations were selected for further validation and when conservation was authenticated, mutations located in the mid-section of the gene sequence were selected, due to the increased stability of gene structure and lower susceptibility to alignment artifacts.

### Gene Expression Analysis of Elovl3 and Abcc11 in Pangolin Tissues

RNA-seq data for the tissues of *M. javanica* from previously published bioprojects were downloaded from NCBI’s database (Online Resource 2). In order to map the SRAs to the reference genome, we indexed the genome using Hisat2 v.2.2.0 (Kim et al. 2015, 2019; Zhang et al. 2021) to enable the mapping of both forward and reverse sequences (within the SRA archives) to the species’ reference genome (YNU_ManJav_2.0). In this mapping, the option for downstream transcriptome assembly was triggered to reduce computational and memory consumption with transcript assembly. The generated SAM files were then converted and sorted to BAM files using samtools and submitted to featureCounts, to quantify the raw reads that were mapped to the transcriptomic components (Liao et al. 2014); the raw read quantification was then transformed to transcripts per million (TPM) using an in-house script.

## Results and Discussion

**Fig. 2:**
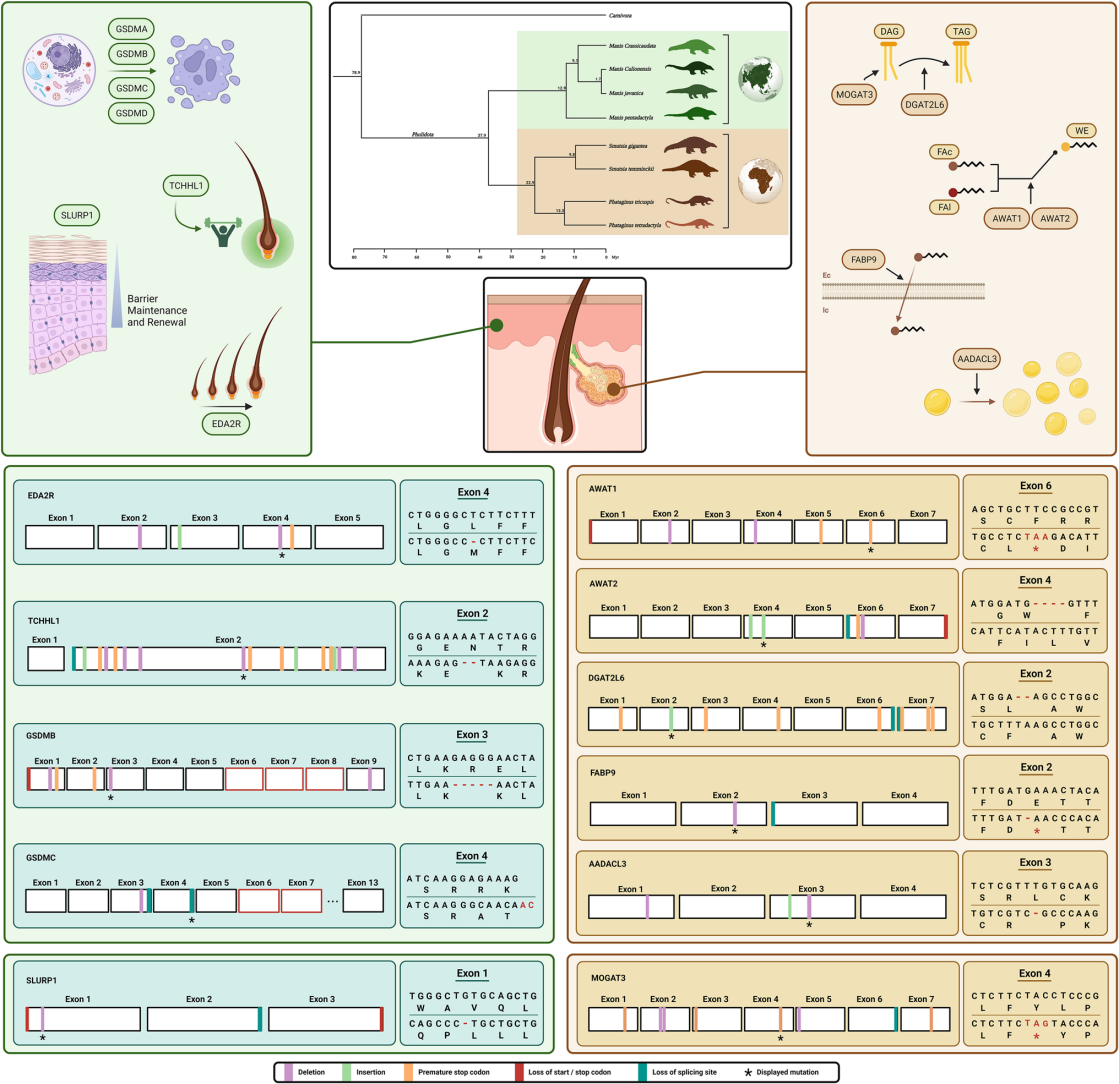
Mutational landscape of skin-specific genes in pangolins. Central top panel shows a phylogenetic tree of the evolution of Pholidota and geographic distribution of extant species. On the upper half, in each side are represented the gene pathways: on the right side, genes associated with the production of sebum and lipid synthesis; on the left side, genes which play a role in skin defense, integrity of skin layers, and homeostasis. On the lower half of the panel a representative and conserved (within pangolin lineages or in stem of the pangolin clade) disruptive mutation for each gene is shown; as well as a more global view of the numerous non-synonymous modifications along the gene. For *Slurp1* and *Mogat3*, no fully conserved mutations were detected, therefore the mutations displayed in the corresponding box are from *P. tricuspis*. Created with BioRender.com

In the present work, we set to analyze the status of skin-specific genes, mostly involved in sebaceous gland function, across four Pholidota species: *M. javanica*, *M. pentadactyla*, *M. crassicaudata*, and *P. tricuspis*. Pangolins diversified approximately 38 million years ago and have since colonized two continents, Africa and Asia (Fig. 2) (Gaubert et al. 2018). We first identified the gene-containing scaffold in each of the investigated genome assemblies, using *PseudoIndex* to classify the coding condition of the studied genes in pangolin genomes (Alves et al. 2020) (Online Resource 3). This classification system varies in a discrete scale from 0 to 5, with 0 suggesting full functionality of the candidate gene and a value of 5 indicating a complete inactivation (Table 1). Genes with *PseudoIndex* above 2 were further validated and the retrieved mutations carefully annotated.

### Sebum-Producing Genes Show Signs of Erosion

Sebum comprises a complex lipid mixture. The biosynthesis of sebum components involves the action of various key modules: i.e., monoacylglycerol O-acyltransferases—*Mogat2* and *Mogat3*; diacyl-glycerol O-acyltransferases—*Dgat2* and *Dgat2l6*; and wax alcohol acyltransferases—*Awat1* and *Awat2* (Bell and Coleman 1980; Turkish et al. 2005; Holmes 2010; Kawelke and Feussner 2015). Besides fatty acid esterification to produce triglycerides or waxes, these modules also encompass fatty acid elongation (i.e., elongases—*Elovl3*), as well as trafficking and signaling (i.e., fatty acid-binding protein—*Fabp9*), required for the upstream regulation of sebum production via fatty acid-responsive transcription factors (i.e., Peroxisome proliferator-activated receptors—PPARs) (Fig. 2) (Trivedi et al. 2006; Kobayashi and Fujimori 2012).

Our comparative analysis showed that numerous disruptive mutations are present in the sebum production-related genes in pangolins. Regarding *Awat1*, using the reference genome we identified and validated, with independent SRA data, a conserved premature stop codon in the sixth exon in *M. javanica*, *M. pentadactyla*, and *P. tricuspis* (Fig. 2; Online Resources 3 and 4); in the *M. crassicaudata* genome, no *Awat1*-containing scaffold was found yet, a possible problem in the genome assembly cannot be discarded (not shown). Other mutations, including nucleotide deletions and premature stop codons, were also retrieved, notably a set of mutations conserved within the *Manis* genus (Online Resources 3 and 4). Curiously, no Awat1 was found in the novel non-annotated genome of M. javanica (GCA_024605085.1), whereas for M. pentadactyla the additional genome largely confirmed previous observations (GCA_024244205.1) (Online Resource 5). Similarly to *Awat1*, *Awat2* displays a mutational pattern concurrent with the inactivation of the gene in the stem of the pangolin clade, exhibiting a conserved loss of a canonical splice site in exon 6 and lack of terminal stop codon in all examined species. Additionally, other disruptive mutations were mapped and validated; notably, a four-nucleotide insertion in the fourth exon found conserved within the *Manis* genus or a 2-nucleotide deletion retrieved in the exon 4 of *P. tricuspis* (Fig. 2; Online Resources 3 and 4). *Dgat2l6* orthologues also displayed several disruptive mutations in pangolins (Fig. 2; Online Resources 3 and 4); yet, unlike *Awat1* and *Awat2*, none was shared across all pangolin species. Still, mutations were found to be conserved within the *Manis* genus (i.e., premature stops codons, nucleotide insertions, and losses of splice sites in multiple different exons), notably a two-nucleotide insertion in the second exon, leading to a premature stop codon, which was further validated by SRA analysis (Fig. 2; Online Resources 3 and 4). In *P. tricuspis*, a set of nucleotide deletions, premature stop codons, and a validated single-nucleotide insertion were identified (Online Resources 3 and 4). Additionally, exon 1 of *P. tricuspis* and exon 5 of *M. pentadactyla* were not found (not shown). Such mutational patterns indicate an independent *Dgat2l6* erosion among pangolin lineages.

We next investigated *Fabp9* (Fatty Acid-Binding Protein 9) and *Aadacl3* (Arylacetamide Deacetylase-Like 3). *Fabp9* is typically expressed in the testis (Selvaraj et al. 2010), but previous findings have also suggested a role in skin homeostasis in Artiodactyls (Jiang et al. 2014). *Aadacl3*, although poorly studied, appears to be related with epidermal fat deposition (Lu et al. 2020; Sweet-Jones et al. 2021). Additionally, analysis of the Human Protein Atlas (www.proteinatlas.org) shows a very specific pattern with expression noted only in skin, breast, and placenta (not shown). Importantly, both these genes have been found to be inactivated on the stem Cetacea branch (Huelsmann et al. 2019; Lopes-Marques et al. 2019a, b; Springer et al. 2021). *Aadacl3* is also eroded in the African elephant (Huelsmann et al. 2019), a lineage where the presence of sebaceous glands has been contentious (Spearman 1970; Lopes-Marques et al. 2019a, b). Sequence analysis allowed the identification of a conserved single-nucleotide deletion in the third exon of *Aadacl3*, found in *M. javanica*, *M. pentadactyla*, and *P. tricuspis*, leading to the emergence of a premature stop codon (Fig. 2; Online Resources 3 and 4), as well as a conserved two-nucleotide insertion in the same exon (Fig. 2; Online Resources 3 and 4). No gene ORF was found for *M. crassicaudata* possibly due to low-quality genome assembly (not shown). Regarding *Fabp9*, a transversal canonical splice site loss was found in exon 3 for *M. javanica*, *M. pentadactyla*, and *P. tricuspis*. Additional lineage-specific nucleotide deletions were also retrieved and validated (Fig. 2; Online Resource 3 and 4). In *M. crassicaudata* the first exon was not found (Online Resources 3). Finally, for *Mogat3*, no conserved mutation was detected, although the genomic sequences display several species-specific mutations across the four species, including a premature stop codon on the sixth exon of *M. pentadactyla*, a single-nucleotide deletion in the fourth exon of *M. crassicaudata* and numerous insertions in the second exon of *P. tricuspis* (Fig. 2; Online Resources 3 and 4). During our analysis, we came across a possible case of gene duplication, followed by erosion of both copies of the gene (not shown), similarly to a previous duplication found in the hippopotamus (Springer et al. 2021). Altogether, our analyses show a comprehensive mutational landscape in sebum-related genes and highlight distinct evolutionary routes, with genes inactivated in the stem of the pangolin clade (i.e., *Awat1*) and genes apparently lost independently in both Asian and African lineages (i.e., *Dgat2l6*).

### Elovl3 is Functional and Expressed in Pangolin Skin Tissue Compartments

**Fig. 3:**
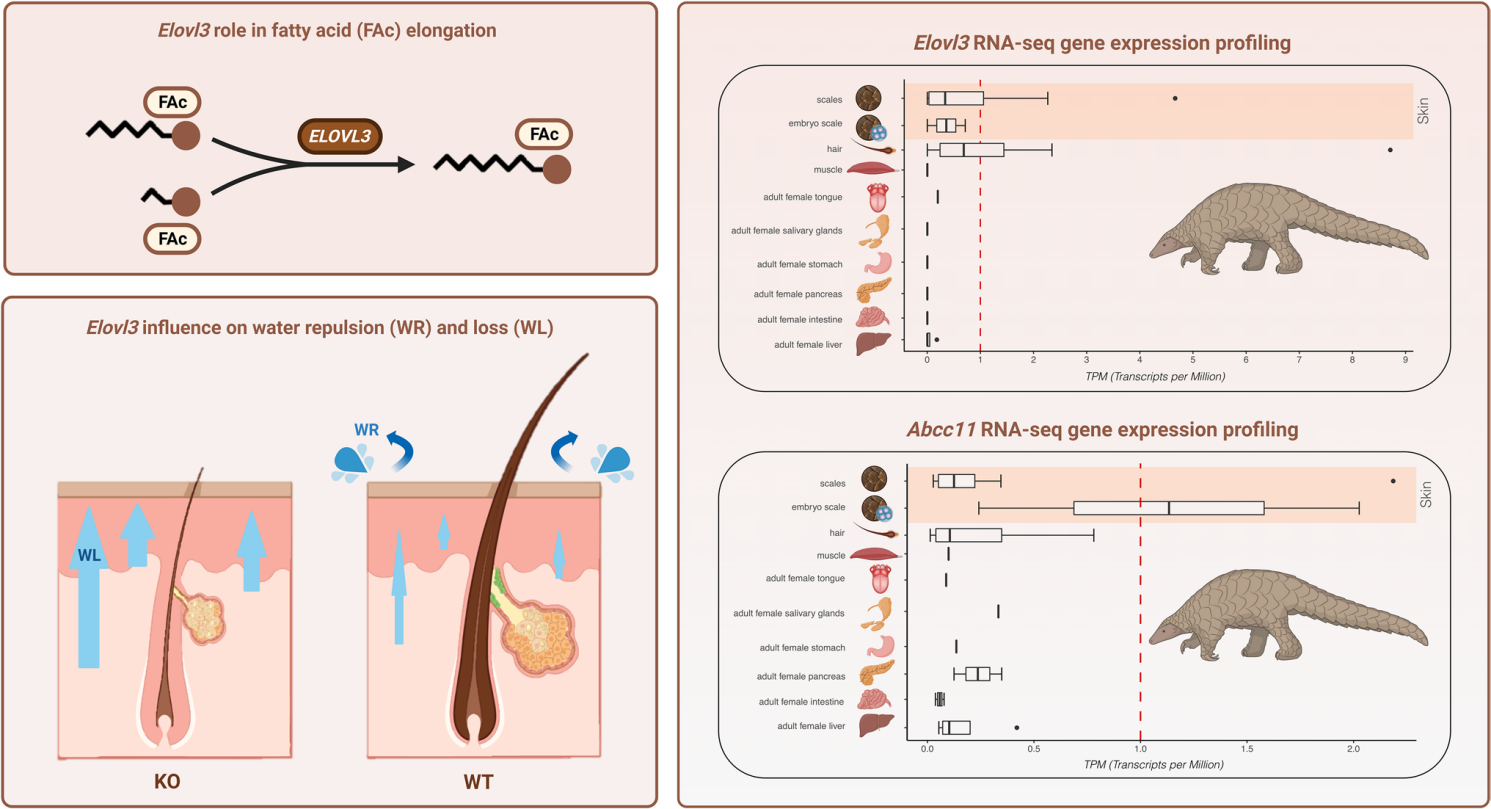
Tissue expression of *Elovl3* and *Abcc1* in the Java pangolin (*M. javanica*)

Fatty acid elongation is a critical pathway for skin lipid homeostasis. A skin-specific elongase, *Elovl3*, has been shown to participate in the formation of specific and essential neutral lipids (Westerberg et al. 2004). In effect, the removal of *Elovl3* in mice leads to a phenotype of sparse hair coat, hyperplastic pilosebaceous unit, and perturbation of hair lipid contents (Westerberg et al. 2004). In Cetacea, *Elovl3* was previously shown to display inactivating mutations (Lopes-Marques et al. 2019a, b; Springer et al. 2021). The identified and examined pangolin *Elovl3* orthologues (Online resource 6) all classified with a *PseudoIndex* of 0, an indication of sequence functionally (Table 1). Yet, we could not exclude deleterious mutations in the regulatory region of the gene that could hamper gene expression and function. Thus, we next examined the expression of *Elovl3* in a comprehensive panel of tissues (Fig. 3; Online Resource 2). Of the examined tissues, the Pholidota *Elovl3* is uniquely expressed in the skin components, including hair follicles (Fig. 3).

### Hair Growth and Robustness are Shaped by Gene Loss Episodes

We next expanded our analysis to a group of genes that play a pivotal role in hair follicle homeostasis: Ectodysplasin A2 Receptor (*Eda2r*) and Trichohyalin-Like 1 (*Tchhl1*). *Eda2r* is a membrane receptor which participates in the regulation of the hair follicle growth cycle (Kwack et al. 2019; Lan et al. 2020; Cai et al. 2021; Font-Porterias et al. 2022). Within the *Manis* genus, we detected a single-nucleotide deletion in exon 2 and a premature stop codon in exon 4 of *Eda2r* (Fig. 2; Online Resources 3 and 4); the latter was validated using independent SRAs (Online Resource 4). For *P. tricuspis*, a 2-nucleotide insertion in exon 3 and a premature stop codon in exon 5 were retrieved, denoting an independent *Eda2r* loss across Asian and African pangolin lineage. Additionally, multiple non-conserved alterations of the nucleotide sequence were identified in all four species, including insertions, deletions, premature stop codons, loss of splicing sites, and missing exons (Online Resource 3).

We next investigated *Tchhl1*, a gene responsible for providing mechanical strength to the hair follicle inner root sheath through keratin intermediate filaments (Makino et al. 2020). Numerous disruptive mutations were retrieved and found conserved among members of the *Manis* genus; a conserved two-nucleotide deletion in the second, and last exon of the gene, was selected for further scrutiny using independent SRAs (Fig. 2; Online Resources 3 and 4). For *P. tricuspis*, no gene remnant was found. Curiously, *Tchhl1*, which is chiefly expressed in the *stratum basale* of the epidermal layer, is also inactivated in cetaceans and hippopotamuses (Springer et al. 2021).

### Erosion of the Gasdermin Gene Repertoire

Gasdermins comprise a protein family involved in membrane permeabilization and pyroptosis, a lytic pro-inflammatory type of cell death leading to the release of intracellular contents (Broz et al. 2019; de Schutter et al. 2021). Importantly, members of this gene family are expressed in the skin (Tamura et al. 2007). *Gsdma* is specifically expressed in human epidermis, hair follicles, and sebaceous glands and was recently shown to be required for epidermal cornification and skin regeneration (Lachner et al. 2017; Huang et al. 2023); in mice, *Gsdma3* mutation leads to alopecia (Runkel et al. 2004). *Gsdmb* and *Gsdmc*, expressed in human keratinocytes, were also suggested to contribute to keratinocyte differentiation and cornification (Lachner et al. 2017). In addition to skin build-up and maintenance, gasdermins, notably *Gsdmb* and *Gsdmd*, were shown to affect T-cell differentiation and function, as well as macrophage infiltration, protecting the skin against bacterial infections and contributing to the pathophysiology of skin diseases, such as psoriasis or skin fibrosis (Liu et al. 2021; Yang et al 2022; Ji et al 2023). Our study of the gasdermin family (*Gsdma*, *Gsdmb*, *Gsdmc*, and *Gsdmd*) revealed some very different scenarios regarding the conservation status of these genes. For *Gsdma*, preliminary *PseudoIndex* analysis suggested pseudogenization for *M. crassicaudata* and *M. javanica* (*PseudoIndex*, 5), while P. tricuspis scored 0, suggestive of an intact gene (Table 1). Further analysis highlighted a missing exon in *M. crassicaudata* (exon 6), out of the eleven that compose the gene (Online Resource 3). Regarding *M. javanica*, the available genomes displayed a single (exon 6) or two missing exons (exons 6 and 7; Online Resource 3). *M. pentadactyla* genomes, however, yielded contradicting results, with the *PseudoIndex* analysis scoring 0 for the gene region extracted from the annotated genome (GCA_014570555.1) and scoring 5 with the novel non-annotated genome (GCA_024244205.1) (Table 1 and Online Resource 3). Manual curation of the putatively pseudogenized *Gsdma* in *M. pentadactyla* unraveled two missing exons, as observed in *M. javanica*, in addition to the loss of a splicing site and a single-nucleotide deletion, both in exon 8, confirming the predicted coding status (Online Resource 3). *Gsdmb*, on the other hand, displayed various mutations conserved across the *Manis* lineage, including a five-nucleotide deletion in the third exon, which was independently validated by SRAs (Online Resources 3 and 4). Other non-conserved mutations were identified and several exons could not be found through our analysis, especially in *P. tricuspis*, for which only two out of the nine exons were retrieved, possibly due to a low-quality assembly (Fig. 2). Yet, the *P. tricuspis* sequence also harbored a disruptive premature stop codon in exon 1, which was further validated (Online Resources 3 and 4). Regarding *Gsdmc*, no gene was found for *M. crassicaudata* and *P. tricuspis* but an assembly artifact cannot be discarded (Online Resource 4). Curiously, for *M. javanica* and *M. pentadactyla*, two *Gsdmc* copies were found in the previous available reference genomes, yet the duplication was not corroborated by the novel genome assemblies (GCA_024244205.1; GCA_024605085.1). Nonetheless, all retrieved copies displayed *PseudoIndex* scores of 5 (Table 1 and Online Resource 7). Further analysis highlighted the transversal absence of exons 6 and 7 (Online Resources 3 and 4). Numerous inactivating mutations were also retrieved, including mutations conserved between the novel genome assemblies of *M. javanica* and *M.* *pentadactyla*, such as a two-nucleotide deletion in exon 3, as well as two losses of splicing sites in exons 3 and 4 (Online Resource 3). For *Gsdmd*, *M. crassicaudata* and *P. tricuspis* yielded *PseudoIndex* values above 2, suggesting gene sequence erosion (Table 1). While *P. tricuspis* revealed a case of exon loss in the sixth exon from a total of ten coding exons, in *M. crassicaudata* the alignment similarity of the sixth exon was particularly low, hindering further conclusions (Online Resources 3 and 4). Regarding *M. javanica* and *M. pentadactyla*, *PseudoIndex* scores differed between genome assemblies, yielding a value of 0, indicating a high level of conservation of the genomic sequence, for the reference genomes (Table 1), and a value of 5, suggestive of pseudogenization, for the non-annotated genomes (GCA_024244205.1; GCA_024605085.1). Regarding the latter, further scrutiny highlighted the loss of exon 6, as observed for the remaining species, and a conserved premature stop codon in the last exon of both species. Finally, although our results support several gene erosion events within this gene family, further clarifications may be required to fully ascertain the gasdermin repertoire in pangolin species, given the observed discrepancies between current genome assemblies.

### Skin-Layer Integrity Genes Display ORF-Disruption Mutations

Next, we investigated a gene related to the integrity of the skin layers—Secreted LY6/PLAUR Domain Containing 1 (*Slurp1*)—responsible for the stabilization of epithelial cell junctions (Campbell et al. 2019; Okamoto et al. 2020) and found to be eroded in Cetacea (Themudo et al. 2020). Mutations in this gene underlie a rare palmoplantar keratoderma exhibiting increased keratinocyte proliferation, lipid accumulation, and water barrier deficiency (Fischer et al. 2001). We were unable to identify the *Slurp1*-containing scaffold in the *M. crassicaudata* genome (not shown). The remaining species showed solid evidence of pseudogenization: missing exons, in *M. javanica* and *M. pentadactyla*, loss of splicing sites in *M. pentadactyla*, or a deletion in the first exon of the gene in *P. tricuspis* (Fig. 2). Validation of the mutations using independent SRAs was only possible for *P. tricuspis* (Online Resource 3 and 4).

### Sweat Gland Gene Marker are Functional in Pangolins

Water evapotranspiration from the skin is fundamental for thermoregulation. This physiological process is dependent on the action of a subset of skin elements and the sweat glands: eccrine, opening directly into the skin surface, and apocrine, opening into the pilosebaceous unit (Kobielak et al. 2015). Similarly to sebaceous glands, the presence of sweat glands in pangolins was so far unreported (Liumsiricharoen et al. 2008). Here, we investigated the expression status of *Abcc1*, a gene maker of apocrine sweat glands (Martin et al. 2010). *Abcc1* gene was previously shown to be eroded in Cetacea, paralleling sweat gland loss in this lineage (Oh et al. 2015). Expression analysis revealed a marked gene expression in the skin (Fig. 3). Thus, despite the apparent absence of sweat glands, *Abcc1* was found intact in pangolins.

### Gene Loss and the Uniqueness of the Skin Phenotype in Pangolins

Comparative genomics is a powerful tool to decipher the origin and loss of phenotypic variations (Huelsmann et al. 2019; Zoonomia Consortium 2020; Alves et al. 2021; Fuchs et al. 2022; Zheng et al. 2022). Specifically, gene loss-aware research is reverberating, highlighting the role of secondary losses in the emergence of diverse biological features, including the simplification of body plans (i.e., urochordates), the deconstruction of the vertebrate organs (i.e., stomach, pineal gland), the modulation of sensory acuity (i.e., vision, taste), or even behavior and locomotion (Castro et al. 2014; Zhao et al. 2015; Lopes-Marques et al. 2019a, b; Valente et al. 2021a, b; Carneiro et al. 2021; Ferrández-Roldán et al. 2021; Indrischek et al. 2022).

**Fig. 4:**
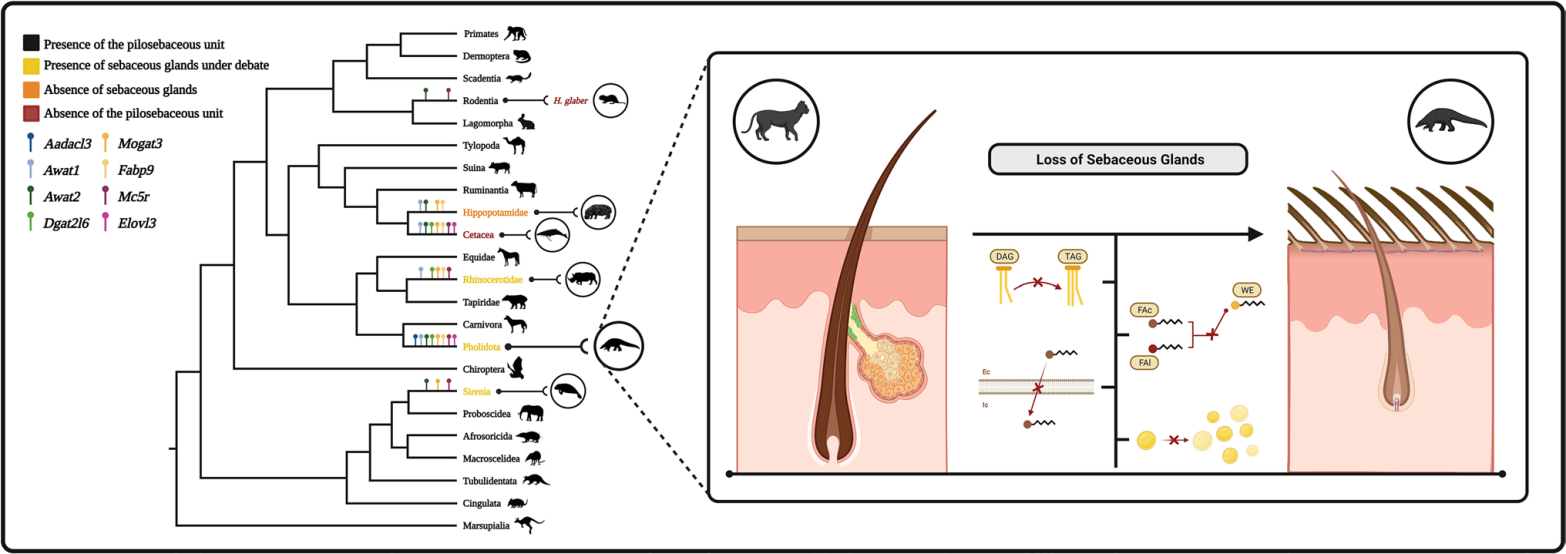
Schematic illustration of the loss of sebaceous glands in mammals and associated gene loss events. Phylogenetic tree of mammals, with emphasis on the different sebaceous gland-related phenotypes. In each branch of the tree are highlighted the different genes each mammalian group lost during the course of its evolutionary development. Each skin phenotype and gene are colour-coded. Created with BioRender.com

Pholidota skin is unique among mammals (Yan et al. 2023). This group evolved from a common ancestral skin phenotype to armored keratinous-scale appendices (Meyer et al. 2013). The main function of the keratinous scales is to serve as protection for the soft skin underneath, making it a natural shield against predators and external harm, such as UV radiation, as well as pathogenic agents (Wang et al. 2016). Keratins are the key components of pangolin scales (Ehrlich et al. 2019). In effect, a recent genome analysis identified a unique expansion in the number of high glycine-tyrosine keratin-associated proteins (HGT-KRTAPs) specifically associated with the phenotype of the pangolin scale (Yan et al. 2023). Interestingly, the presence and functional status of sebaceous and sweat glands in Pholidota are so far unclear (Liumsiricharoen et al. 2008). Previous works emphasized gene loss landscapes in other mammalian lineages with divergent skin phenotypes, such as cetaceans, and large African mammals, such as the African elephant (*Loxodonta Africana*) or the white rhinoceros (*Ceratotherium sinum*) (Fig. 4) (Plochocki et al. 2017; Springer and Gatesy 2018; Lopes-Marques et al. 2019a, b; Springer et al. 2021); this prompted us to address whether similar genomic variations underlined the emergence of the distinctive skin phenotype in pangolins. Our comparative analysis led to the annotation and validation of numerous disruptive mutations in target genes—related with sebum production, skin layer development and maintenance, and hair growth—across four studied species spanning the two pangolin lineages. These results support the role of gene pseudogenization episodes as drivers of the extant skin phenotype in Pholidota. Ancestral (i.e., *Awat1, Awat2*, *Aadacl3*) and independent (i.e., *Dgat2l6*) pseudogenization events in genes associated with epidermal lipids and sebum production strongly suggest a progressive impairment of the sebum-producing molecular machinery in pangolin lineages. Similar genomic signatures were previously proposed for mammalian lineages with derived skin phenotypes, particularly visible in the fully aquatic Cetacea (Sokolov 1982; Springer and Gatesy 2018; Lopes-Marques et al. 2019a, b). Sebum, a mammalian synapomorphy, is mainly produced to serve as a protective layer against UV radiation, bacteria, and skin dehydration (Lobitz 1957; Pappas 2009; Niemann and Horsley 2012). These functions, although vital when considering exposed skin, may have diminished relevance in an armor-like skin, as observed in Pholidota (Fig. 4). A similar hypothesis can be drawn regarding genes related to skin protection against external dangers (i.e., microbial infection)—the gasdermin family, responsible for host defense and cell death (Tamura and Shiroishi 2015). In effect, pangolins display an innate immune gene repertoire that is strikingly variable as compared to other mammalian lineages (Haley 2022), and events of pseudogenization have been described for various genes (e.g., viral DNA sensors cGAS and STING; Fisher et al. 2020).

Whereas sebaceous gland dismantling is supported by our data, the current results do not unequivocally clarify the fate of sweat glands in pangolins. *Abcc1* gene, a marker of apocrine sweat glands (Martin et al. 2010), generally associated with the hair follicle, was shown to be intact and expressed in pangolins. Yet, unlike their back and tail, the abdomen of pangolins yields an exposed skin with sparse hair and thicker *stratum corneum* (Meyer et al. 2013). In agreement, evidence of erosion was found in genes related with keratinocyte proliferation and stabilization (i.e., *Slurp1*) or hair development and mechanical strength (i.e., *Edar2*, *Tchhl1*) (Campbell et al. 2019; Lan et al. 2020; Cai et al. 2021). Conversely, other genomic components were found intact. Among these, we find *Alox15A* and *Alox3*, epidermal lipoxygenases participating in the maintenance of the cornified layer (Krieg et al. 2013) (not shown) or *Elovl3*. Such mosaic gene retention has been previously proposed for mammalian species with derived skin phenotypes (i.e., elephant, rhinoceros, hippopotamuses) (Lopes-Marques et al. 2019a, b; Springer et al. 2021).

## Conclusion

Our findings show that species of the order Pholidota display numerous skin-related gene pseudogenization events paralleled by the dismantling of the sebaceous gland in this mammalian group and the emergence of their idiosyncratic skin phenotype. Importantly, the present work reinforces the role of gene loss as a powerful evolutionary driver, notably in transitional scenarios or radical phenotypic adaptation, as reported for other mammalian groups such as the fully aquatic Cetacea and Sirenia.

## Supplementary Information

Below is the link to the electronic supplementary material.

Supplementary file1 (XLSX 10 KB)

Supplementary file2 (XLSX 40 KB)

Supplementary file3 (XLSX 18 KB)

Supplementary file4 (PDF 7047 KB)

Supplementary file5 (PDF 646 KB)

Supplementary file6 (PDF 258 KB)

Supplementary file7 (XLSX 10 KB)
