# Supplementary material for: Decay of Skin-Specific Gene Modules in Pangolins
Source: J Mol Evol. 2023 May 30;91(4):458–70. doi: 10.1007/s00239-023-10118-z (PMC10277264; doi:10.1007/s00239-023-10118-z)

SRA Validation of an insertion in exon 3 of *Aadacl3*:

Manis javanica:

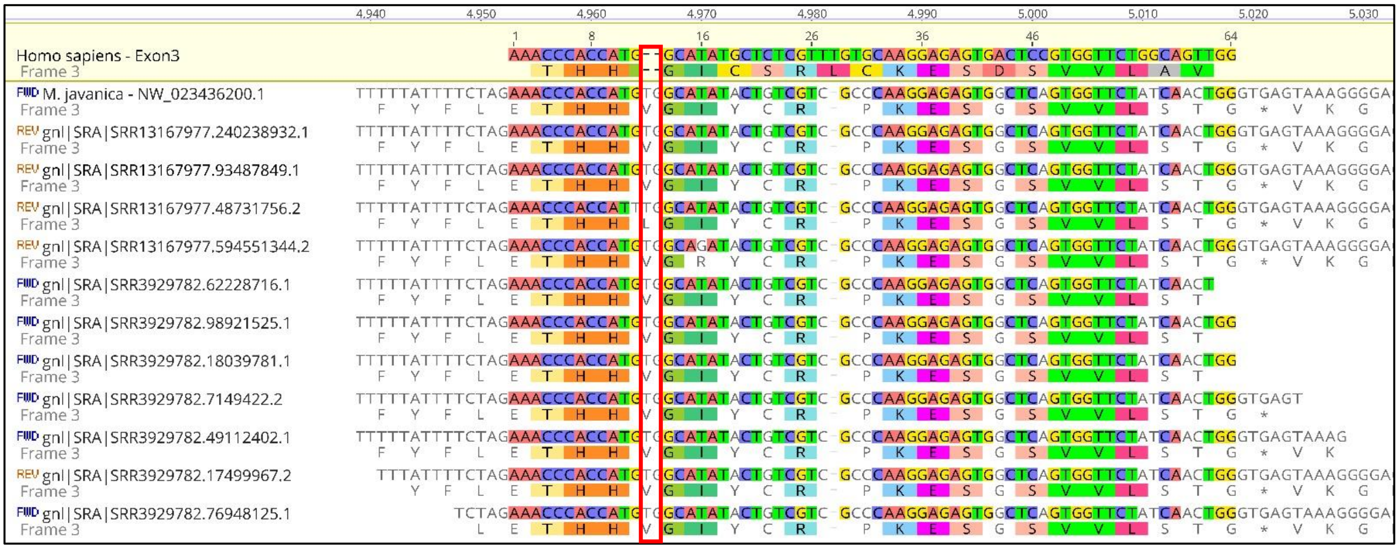

Manis pentadactyla:

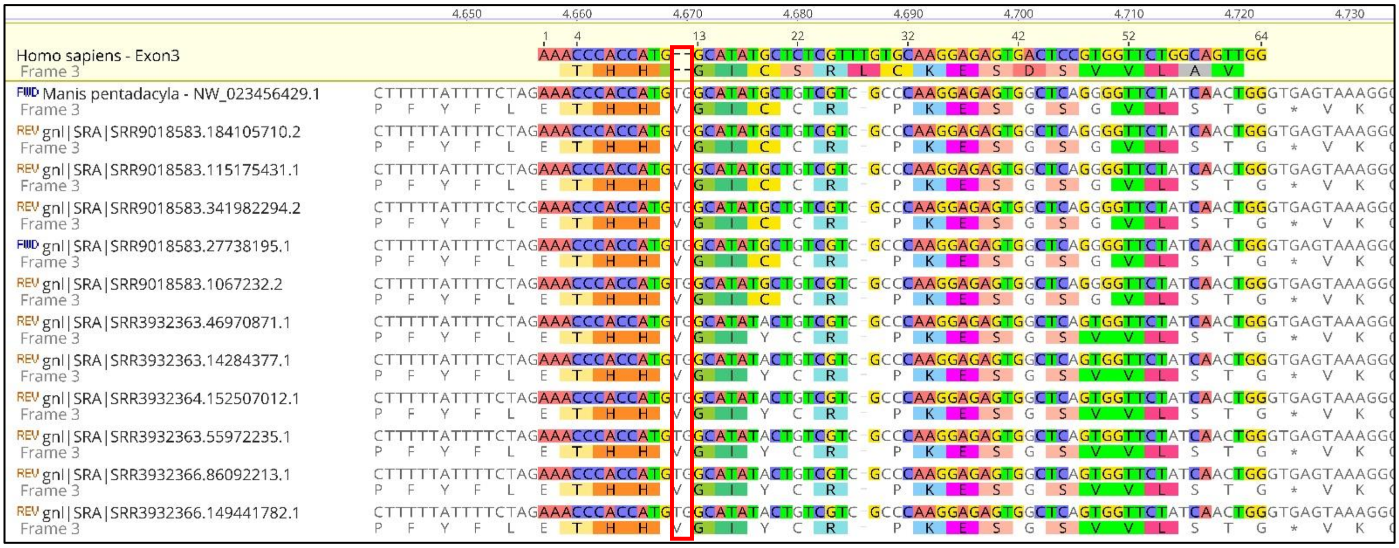

Phataginus tricuspis:

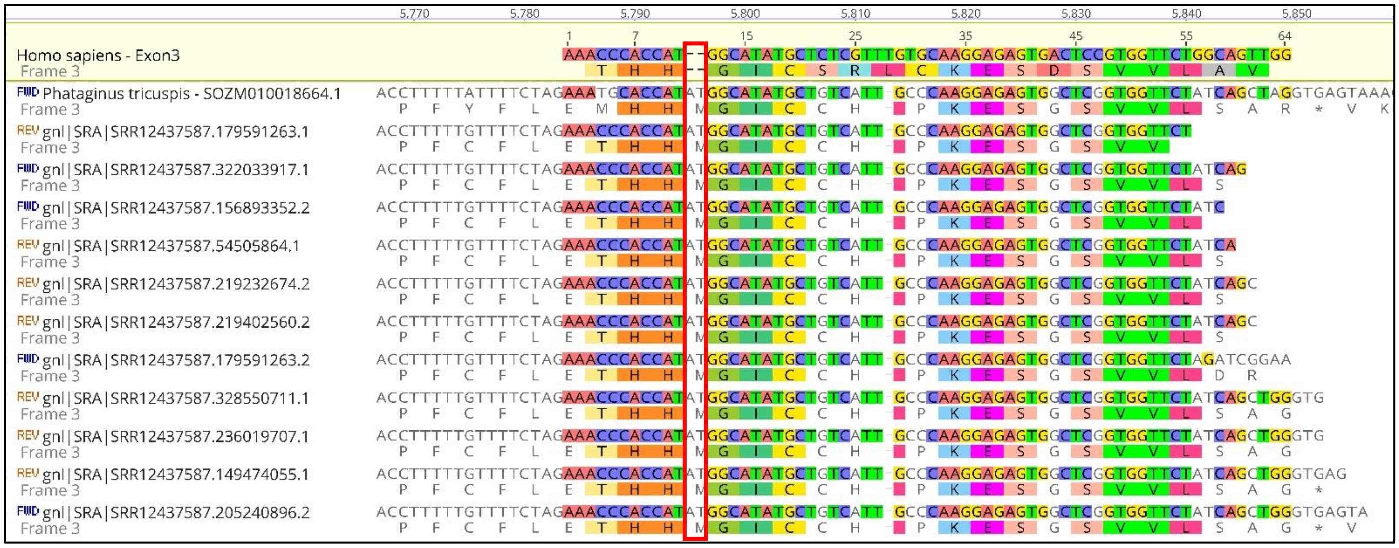

SRA Validation of a premature stop codon in exon 6 of *Awat1*:

Manis javanica:

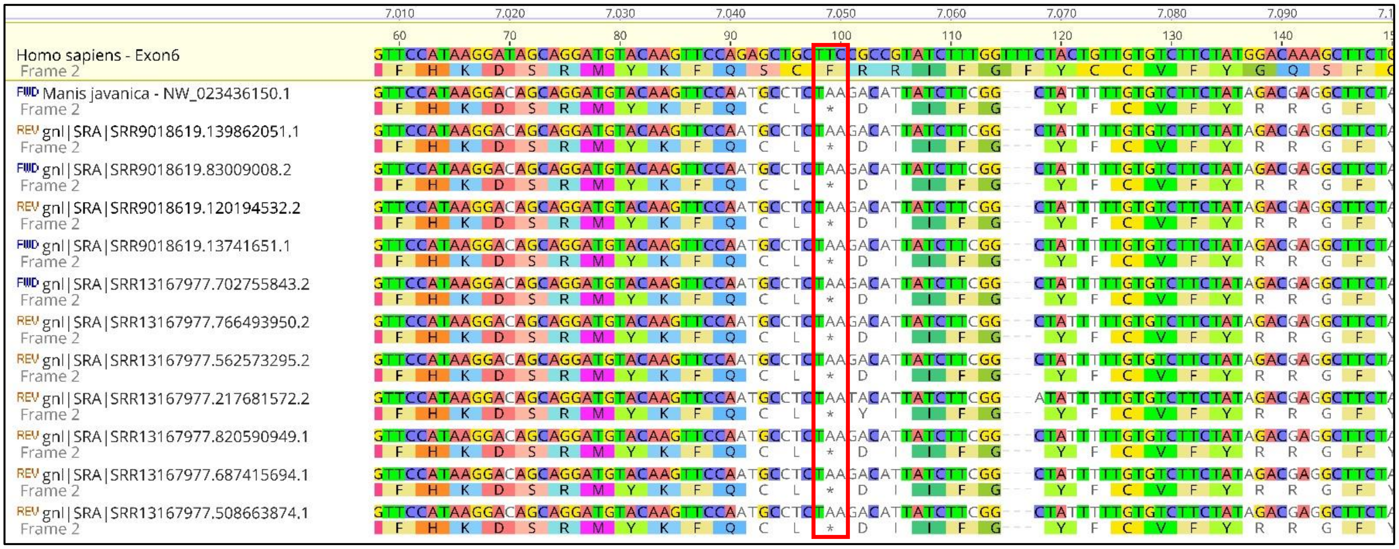

Manis pentadactyla:

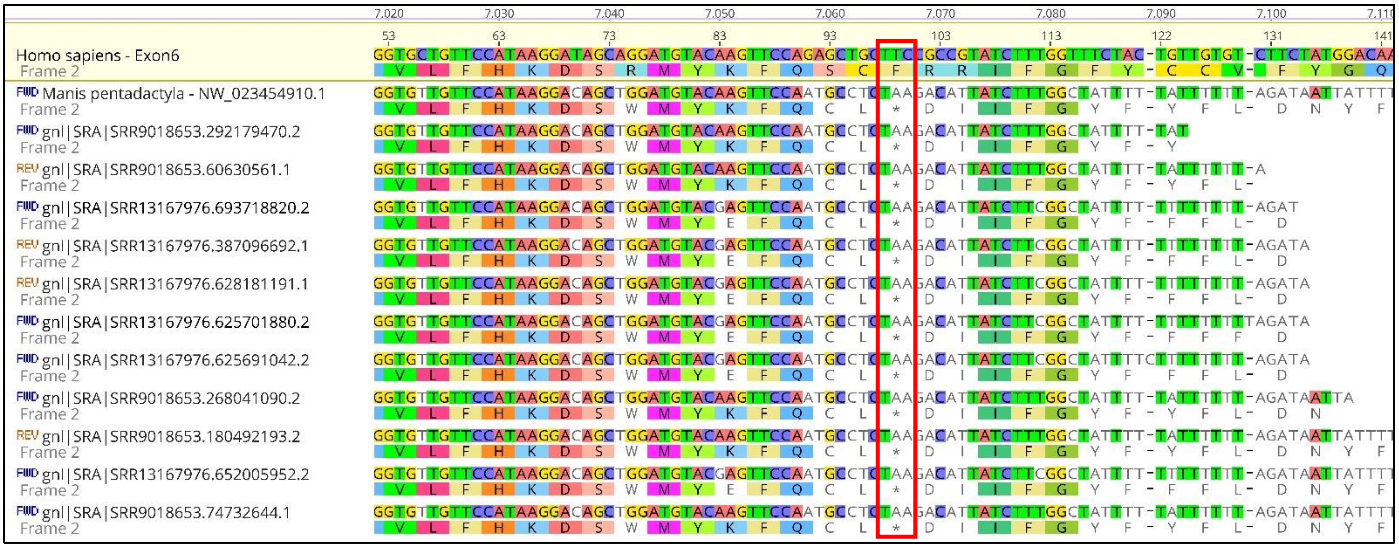

Phataginus tricuspis:

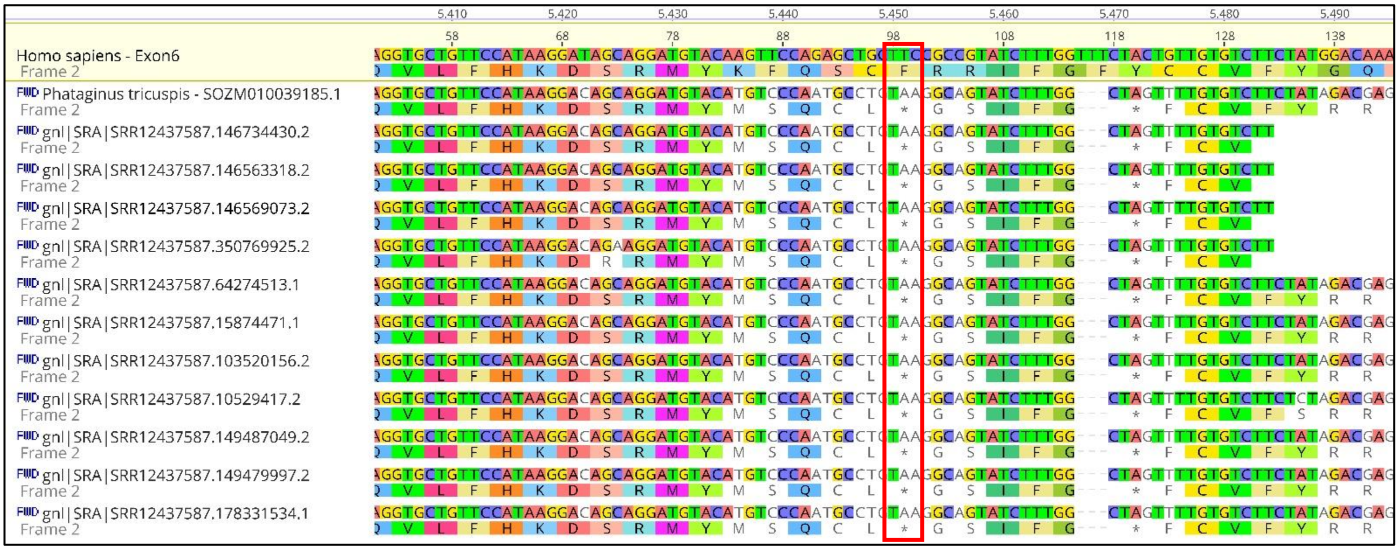

SRA Validation of an insertion in exon 4 of *Awat2* in Manis:

Manis javanica:

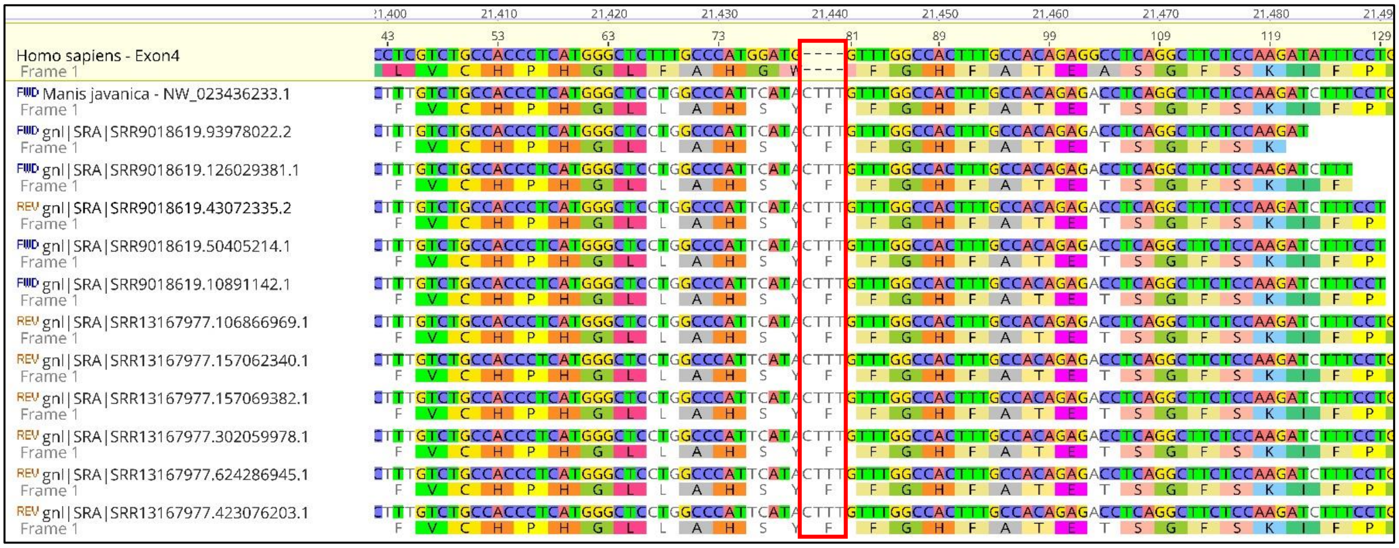

Manis pentadactyla:

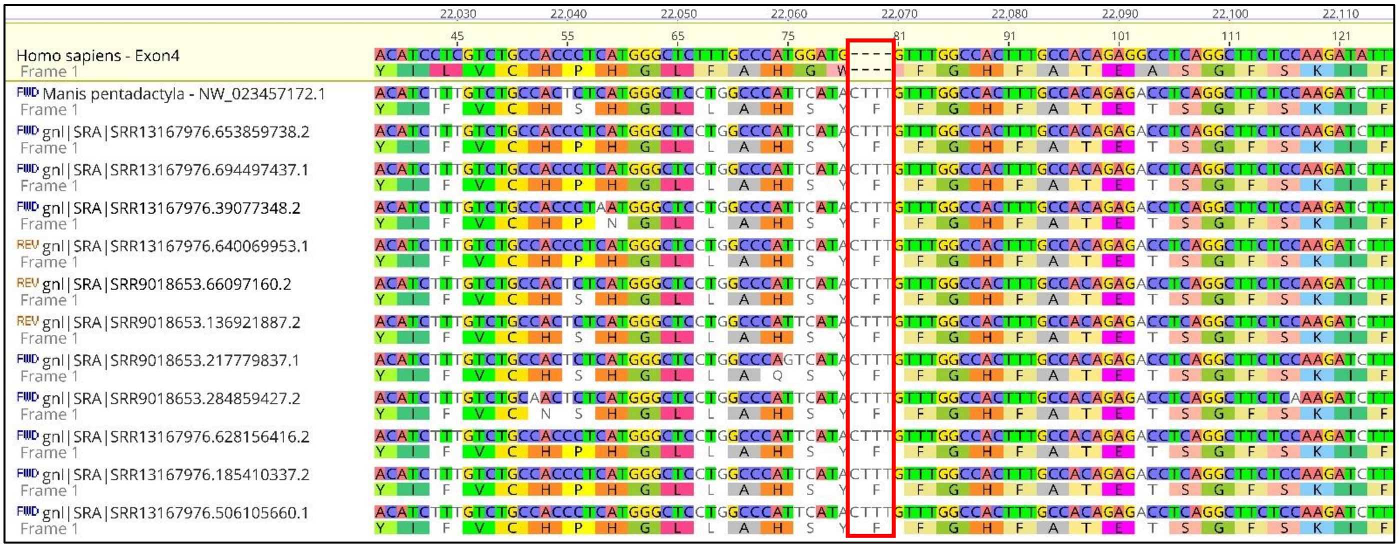

SRA Validation of a deletion in exon 4 of *Awat2* in *Phataginus tricuspis*:

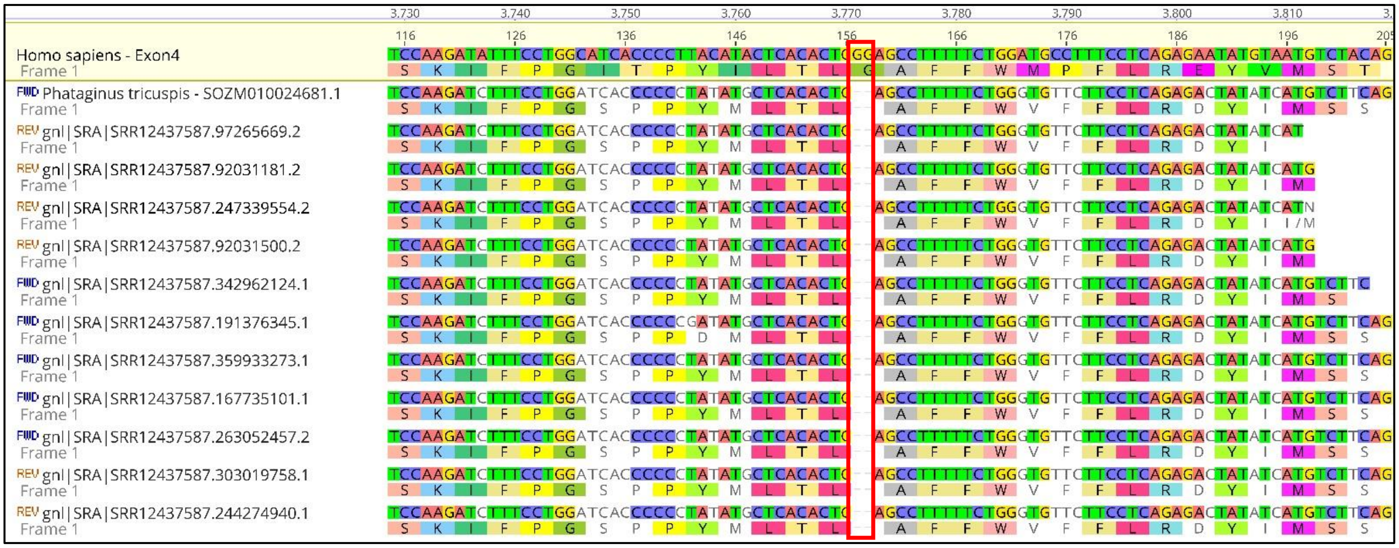

SRA Validation of a deletion in exon 2 of *Dgat2l6* in Manis:

Manis javanica:

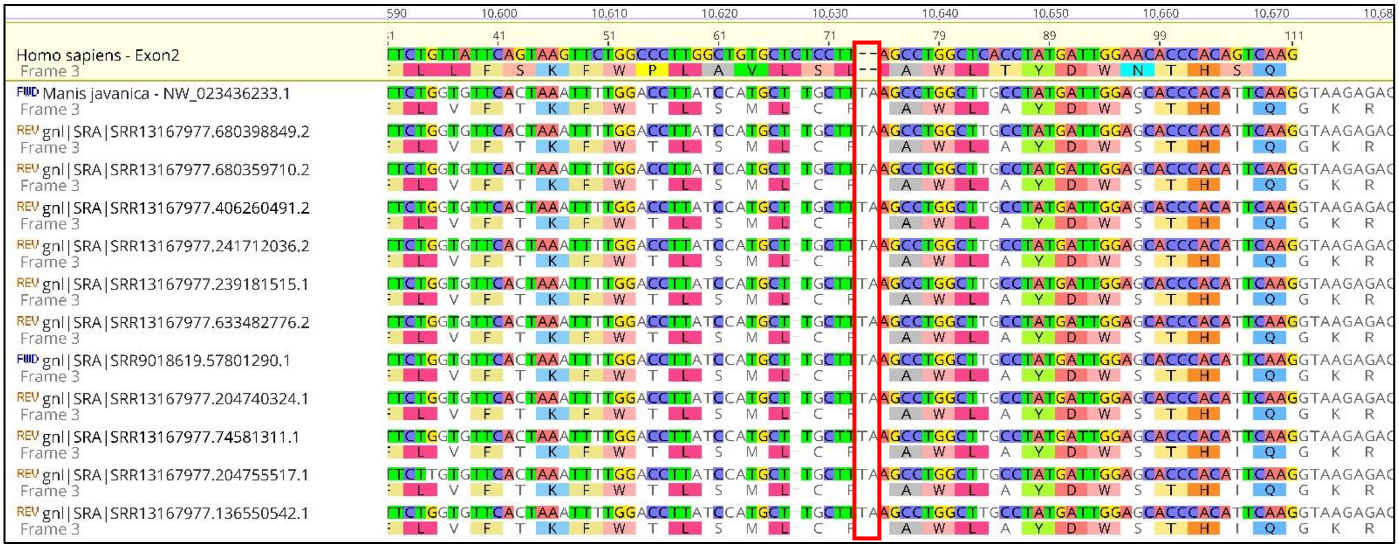

Manis pentadactyla:

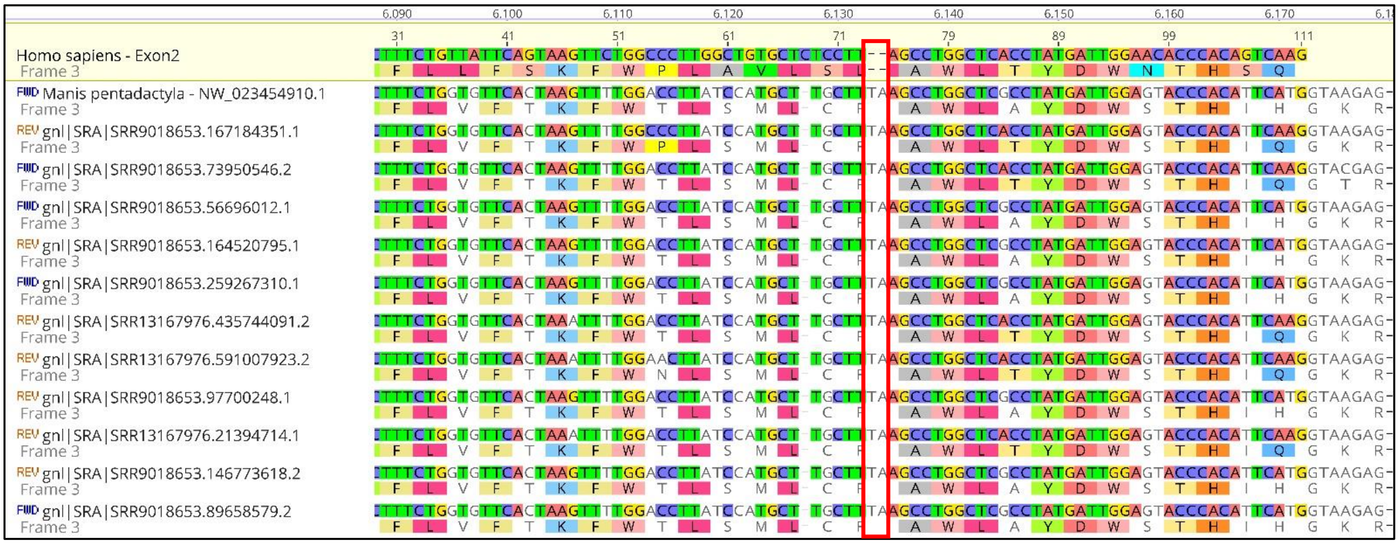

SRA Validation of an insertion in exon 2 of *Dgat2l6* in *Phataginus tricuspis*:

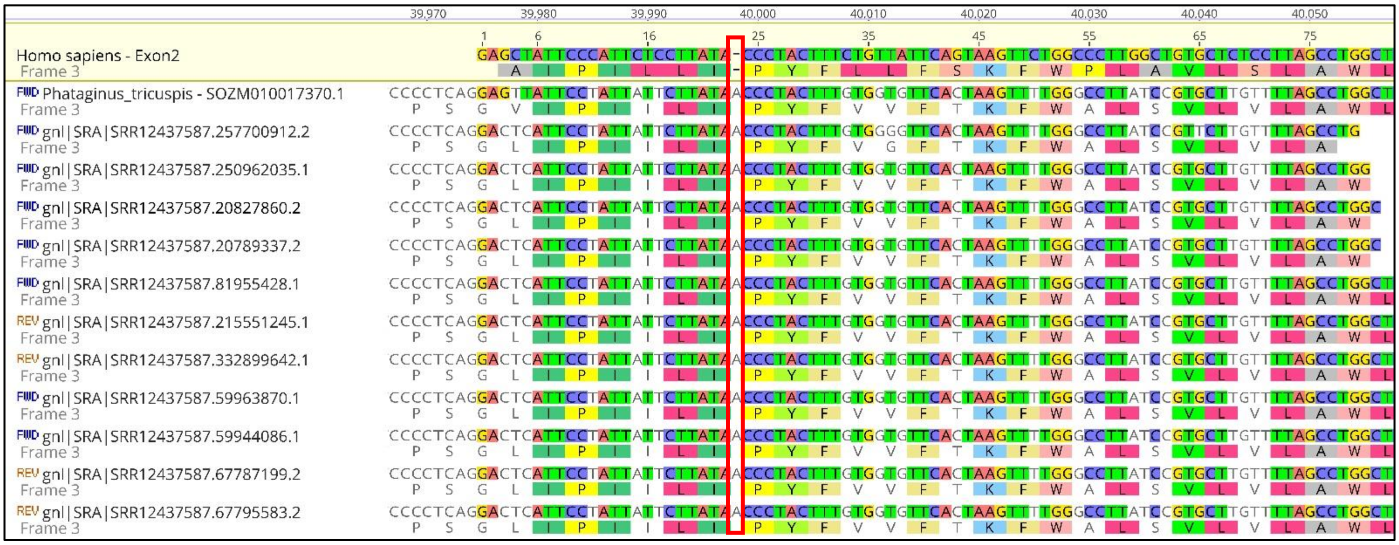

SRA Validation of a premature stop codon in exon 4 of *Eda2r* in Manis:

Manis javanica:

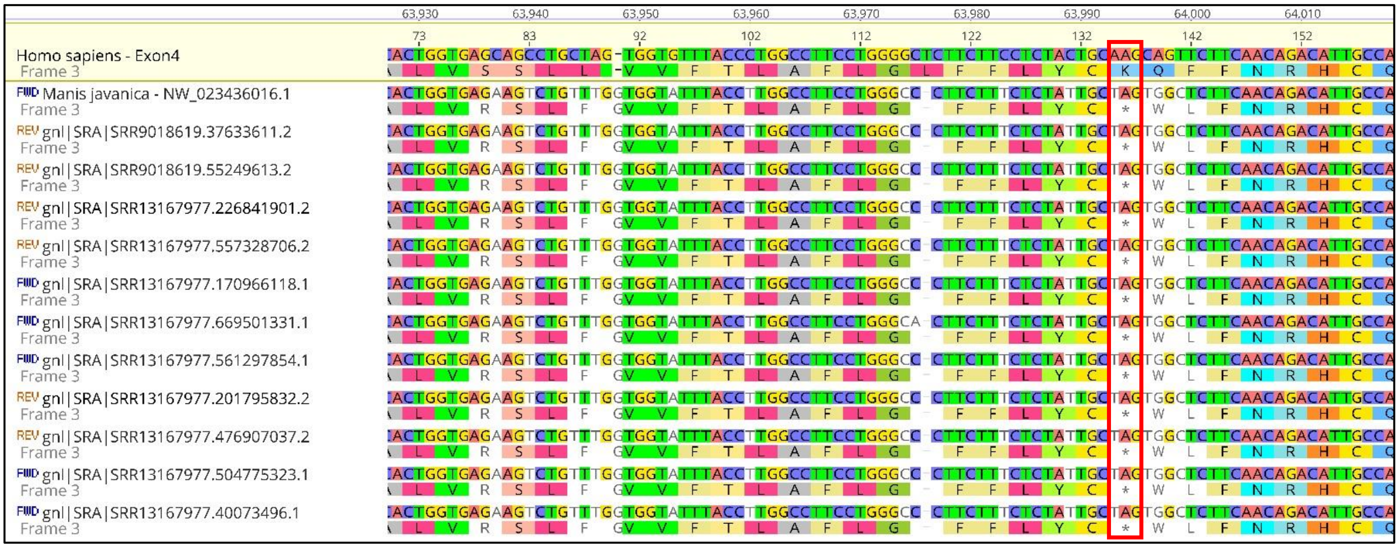

Manis pentadactyla:

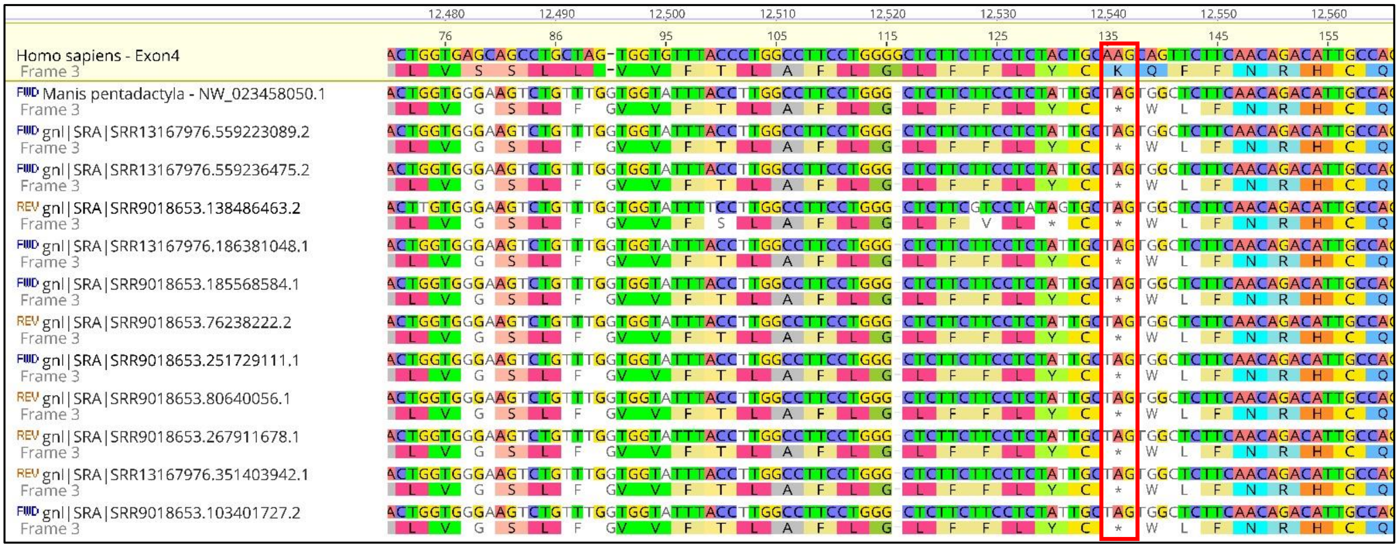

SRA Validation of an insertion in exon 3 of *Eda2r* in *Phataginus tricuspis:*

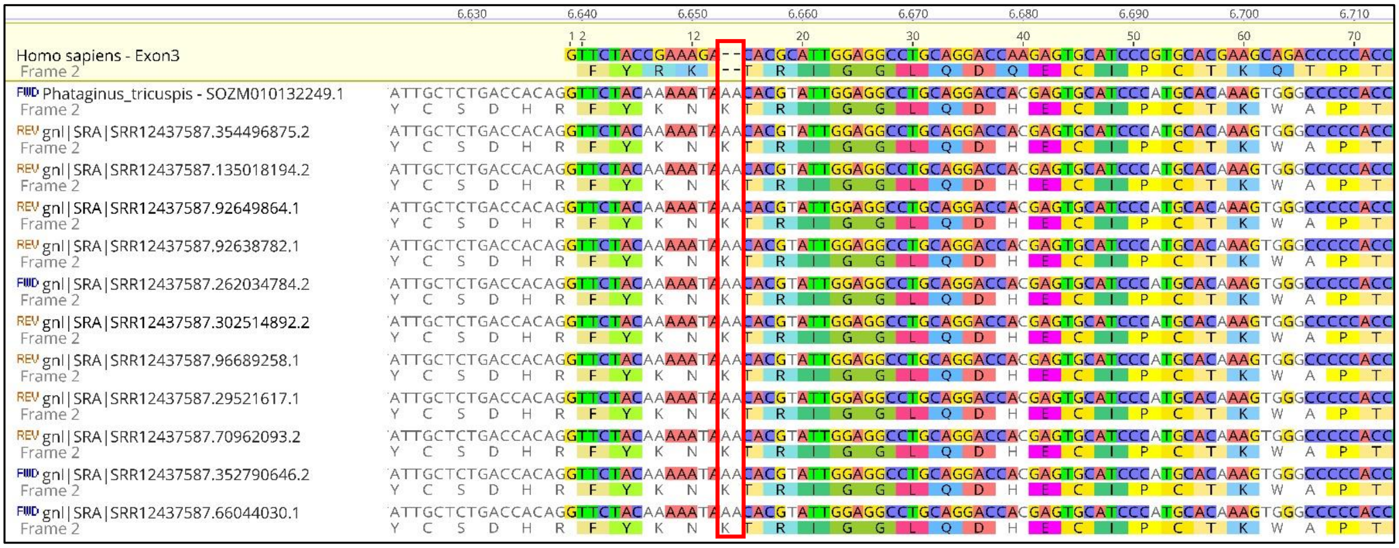

SRA Validation of a deletion in exon 2 of *Fabp9* in Manis:

Manis javanica:

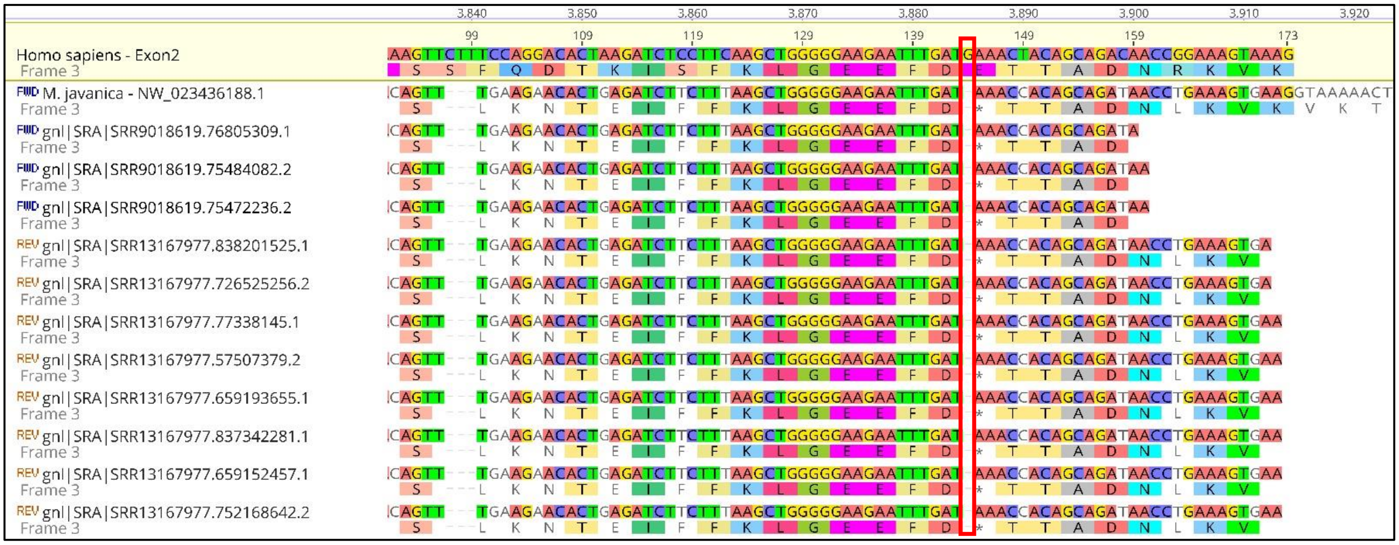

Manis pentadactyla:

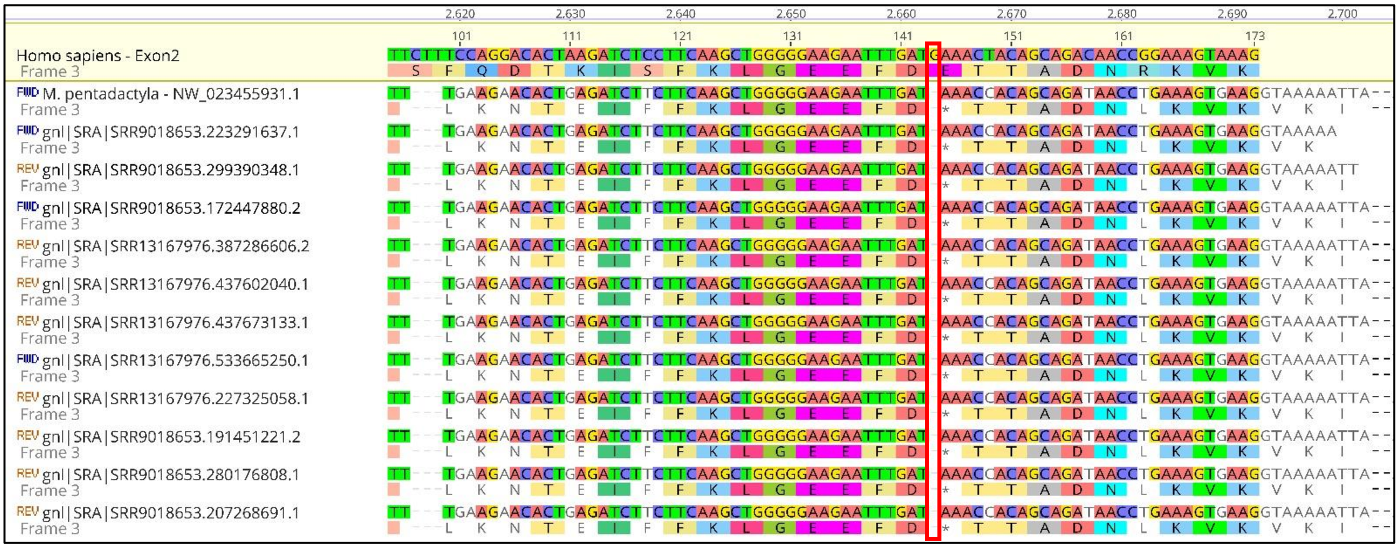

SRA Validation of a deletion in exon 1 of *Fabp9* in *Phataginus tricuspis:*

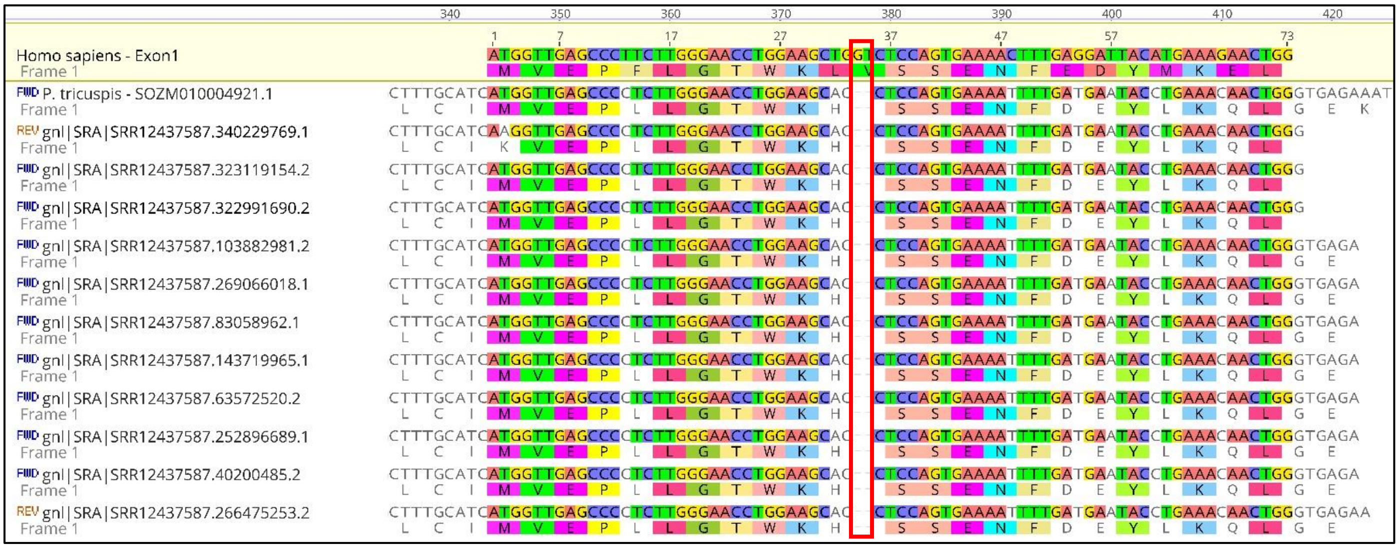

SRA Validation of a deletion in exon 3 of *Gsdmb* in Manis:

Manis javanica:

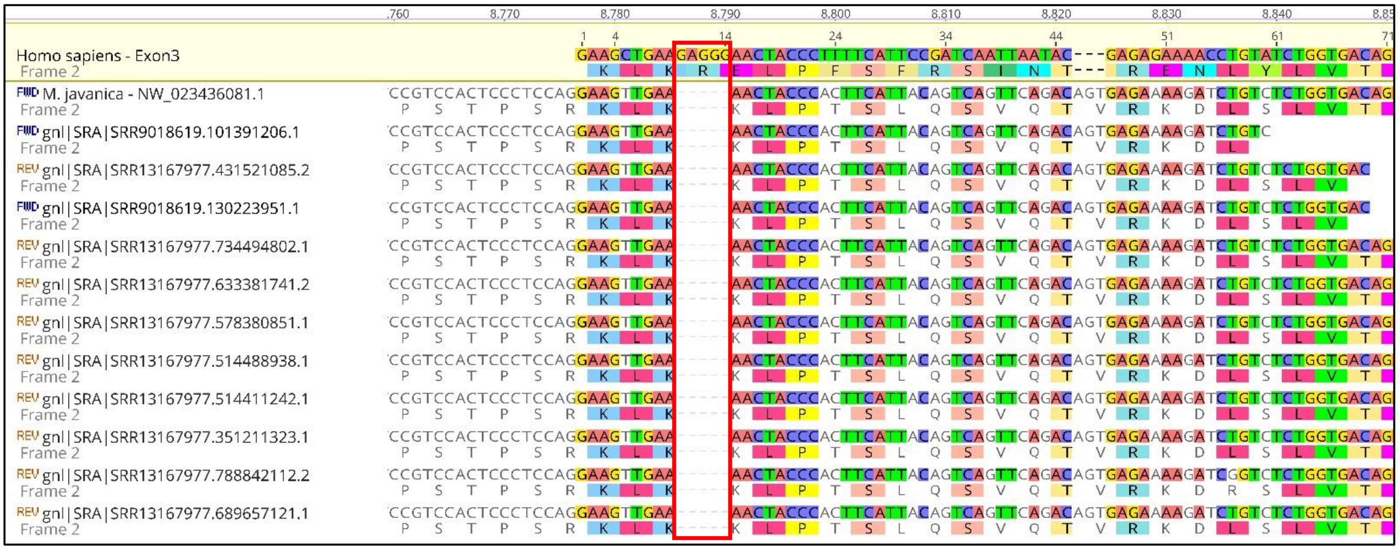

Manis pentadactyla:

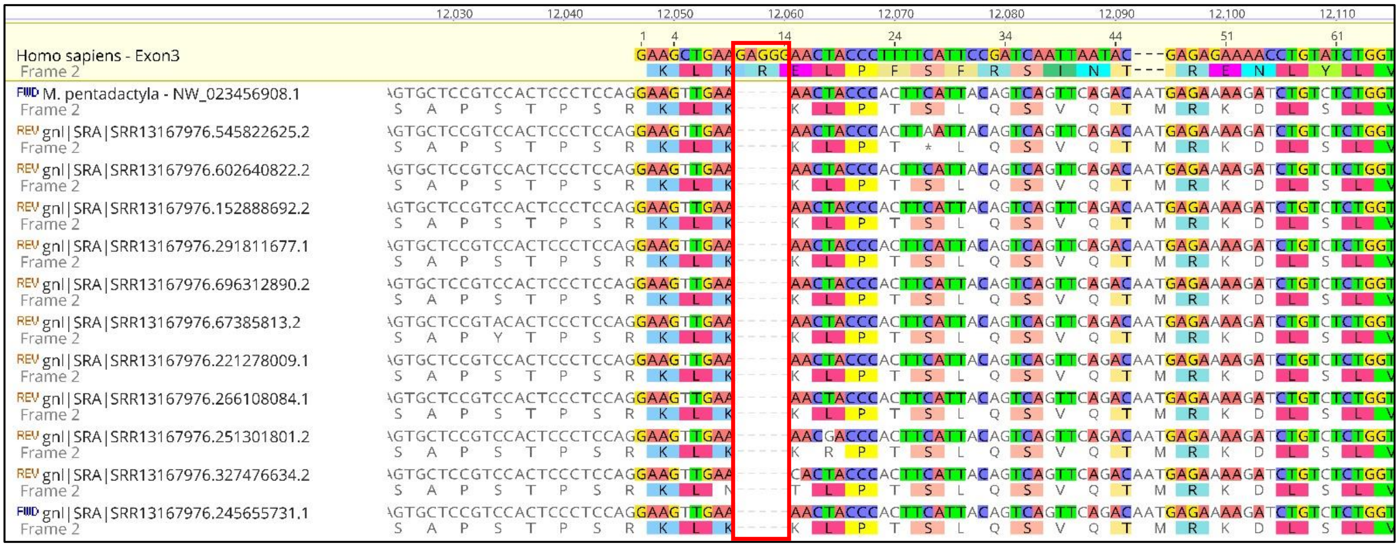

SRA Validation of a premature stop codon in exon 1 of *Gsdmb* in *Phataginus tricuspis:*

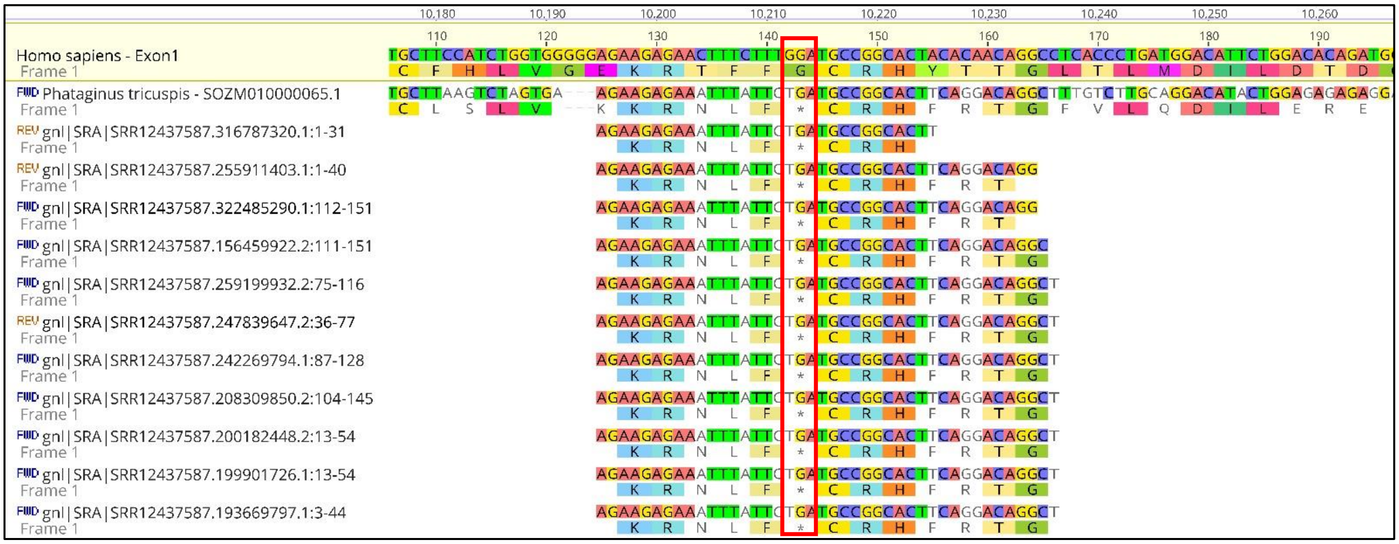

SRA Validation of a premature stop codon in exon 3 of *Mogat3* in *Manis javanica:*

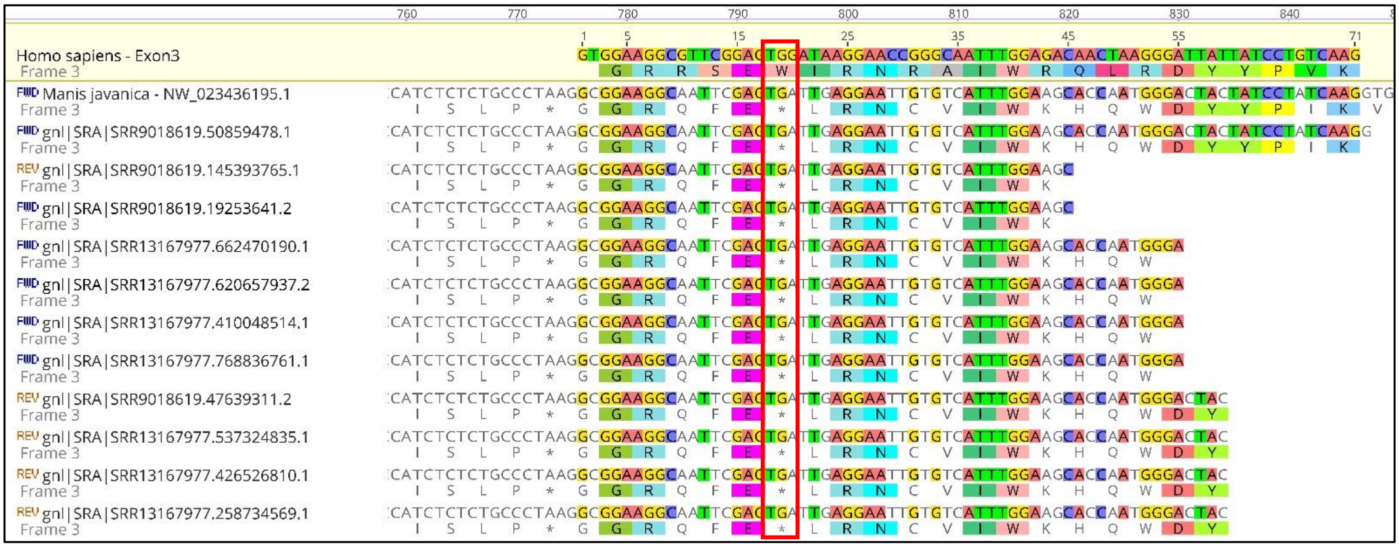

SRA Validation of a premature stop codon in exon 6 of *Mogat3* in *Manis pentadactyla:*

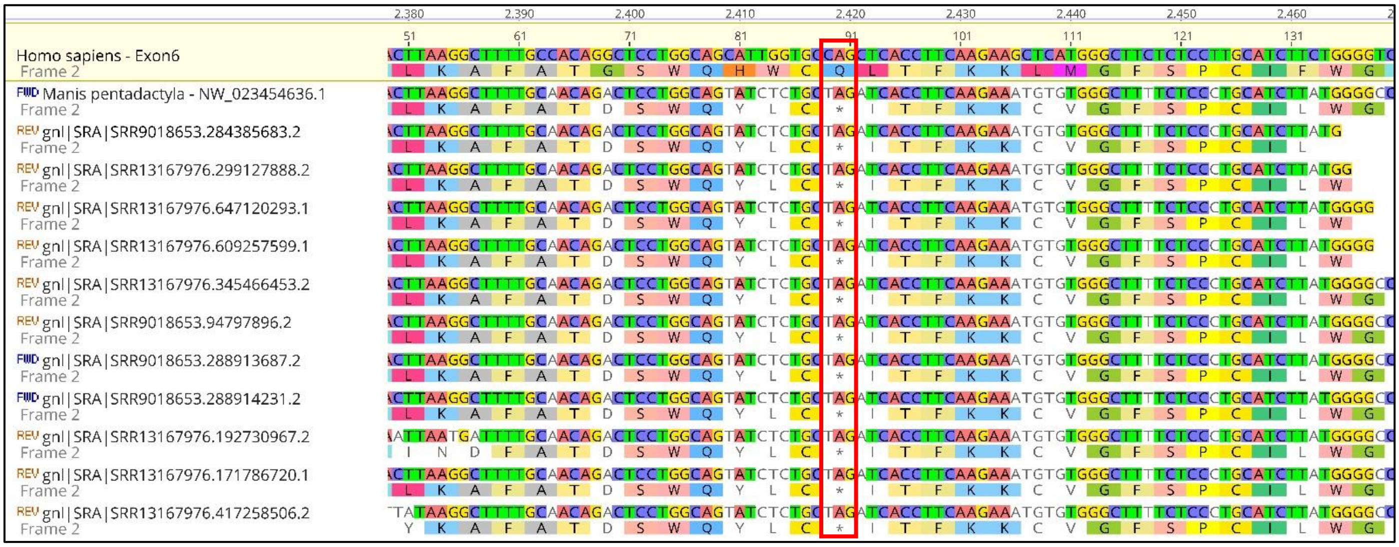

SRA Validation of a premature stop codon in exon 4 of *Mogat3* in *Phataginus tricuspis:*

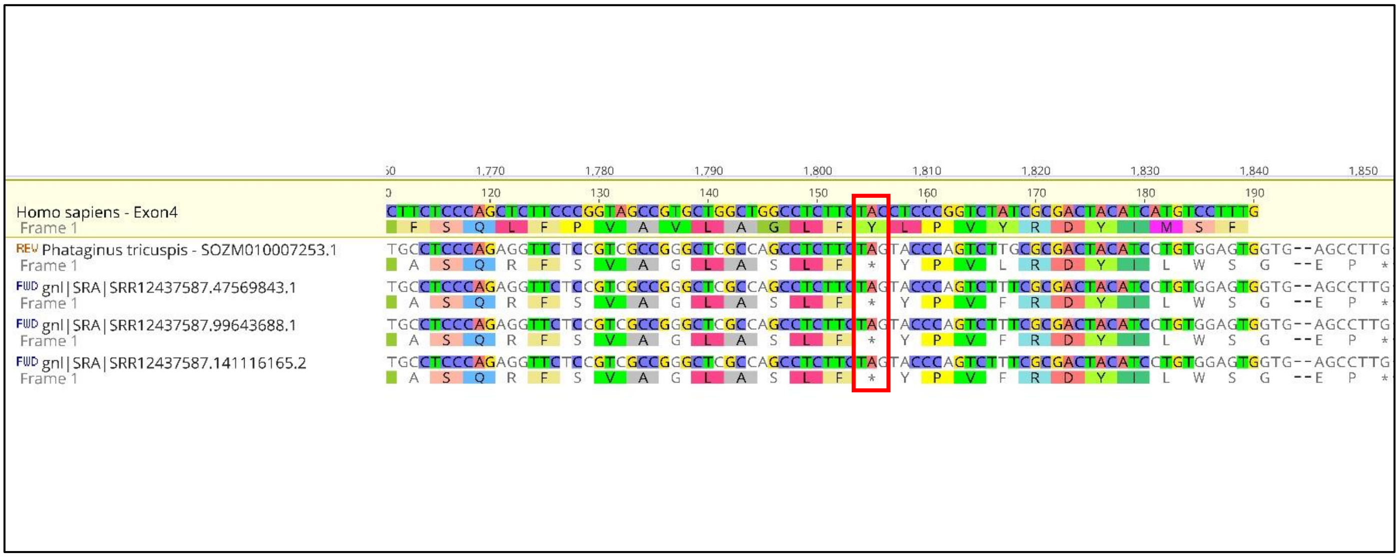

SRA Validation of a deletion in exon 1 of *Slurp1* in *Phataginus tricuspis:*

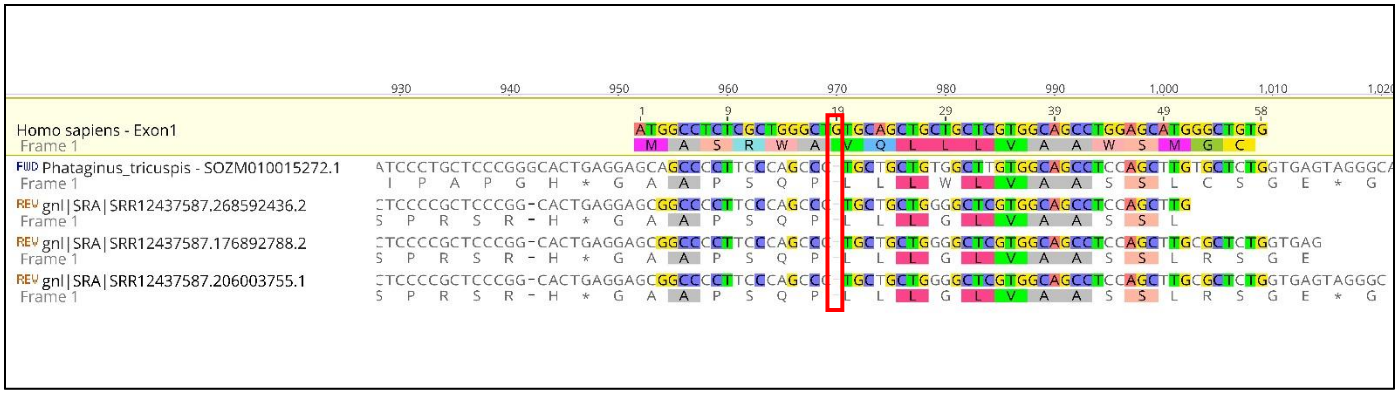

SRA Validation of a deletion in exon 2 of *Tchhl1* in Manis:

Manis javanica:

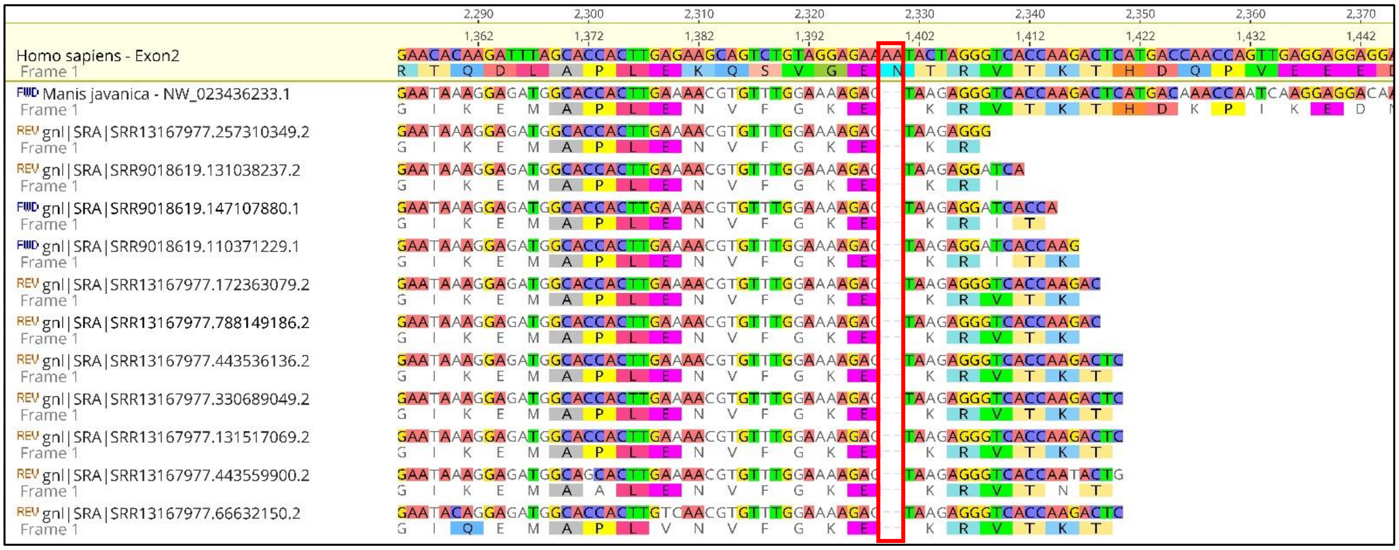

Manis pentadactyla:

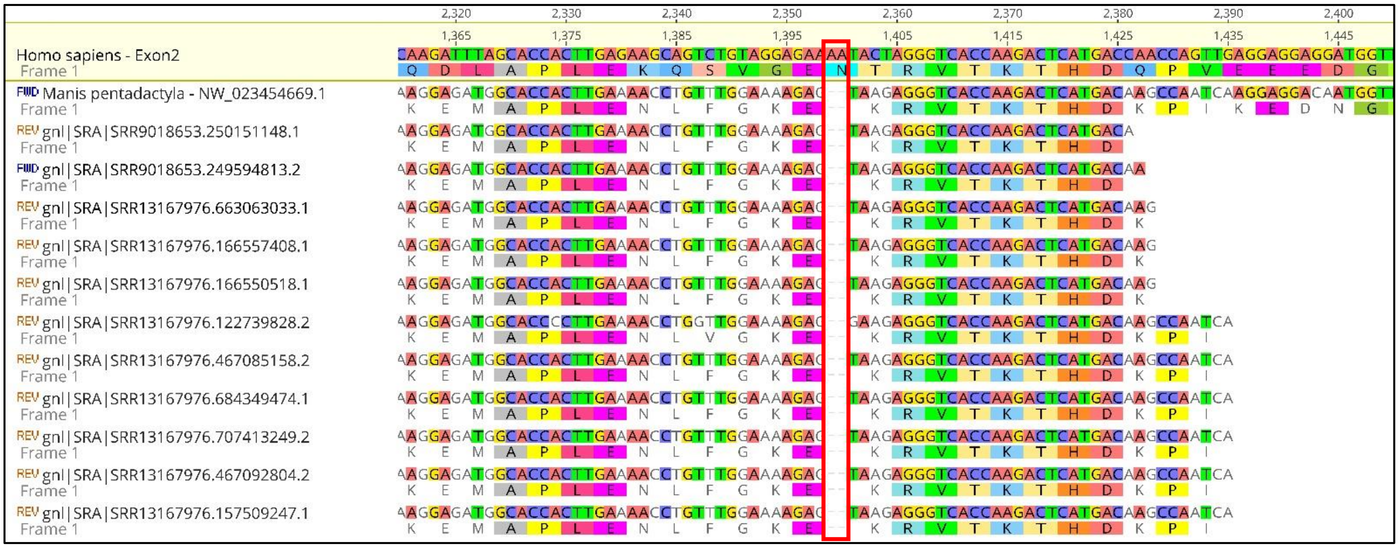

Supplement: Supplementary file 4 — Supplementary file4 (PDF 7047 KB) [file 239_2023_10118_MOESM4_ESM.pdf]
