# Supplementary material for: Decay of Skin-Specific Gene Modules in Pangolins
Source: J Mol Evol. 2023 May 30;91(4):458–70. doi: 10.1007/s00239-023-10118-z (PMC10277264; doi:10.1007/s00239-023-10118-z)
Supplement: Supplementary file 5 — Supplementary file5 (PDF 646 KB) [file 239_2023_10118_MOESM5_ESM.pdf]

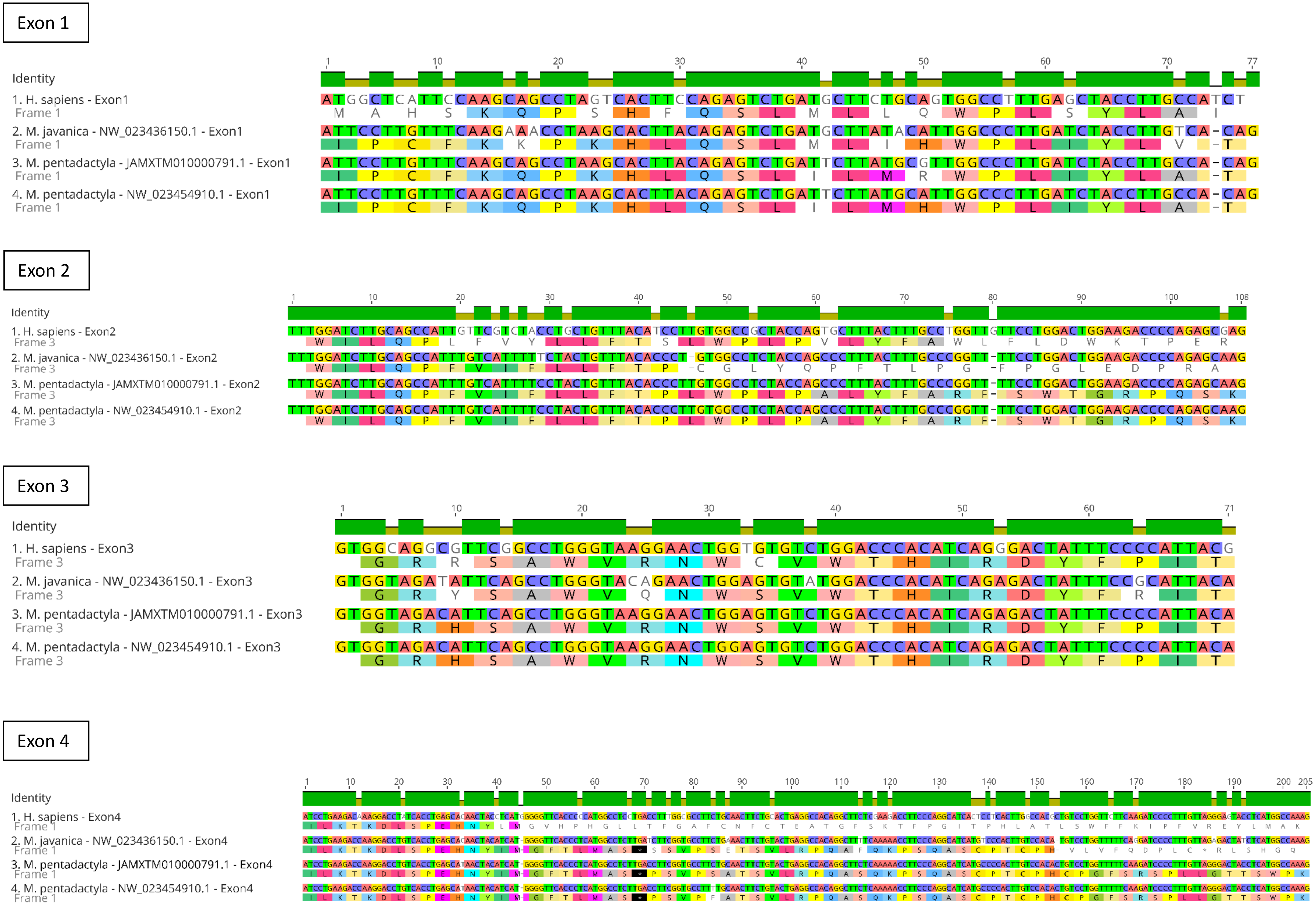
Exon 1
Identity
1. H. sapiens - Exon1
Frame 1
2. M. javanica - NW_023436150.1 - Exon1
Frame 1
3. M. pentadactyla - JAMXTM010000791.1 - Exon1
Frame 1
4. M. pentadactyla - NW_023454910.1 - Exon1
Frame 1
Exon 2
Identity
1. H. sapiens - Exon2
Frame 3
2. M. javanica - NW_023436150.1 - Exon2
Frame 3
3. M. pentadactyla - JAMXTM010000791.1 - Exon2
Frame 3
4. M. pentadactyla - NW_023454910.1 - Exon2
Frame 3
Exon 3
Identity
1. H. sapiens - Exon3
Frame 3
2. M. javanica - NW_023436150.1 - Exon3
Frame 3
3. M. pentadactyla - JAMXTM010000791.1 - Exon3
Frame 3
4. M. pentadactyla - NW_023454910.1 - Exon3
Frame 3
Exon 4
Identity
1. H. sapiens - Exon4
Frame 1
2. M. javanica - NW_023436150.1 - Exon4
Frame 1
3. M. pentadactyla - JAMXTM010000791.1 - Exon4
Frame 1
4. M. pentadactyla - NW_023454910.1 - Exon4
Frame 1

Exon 5

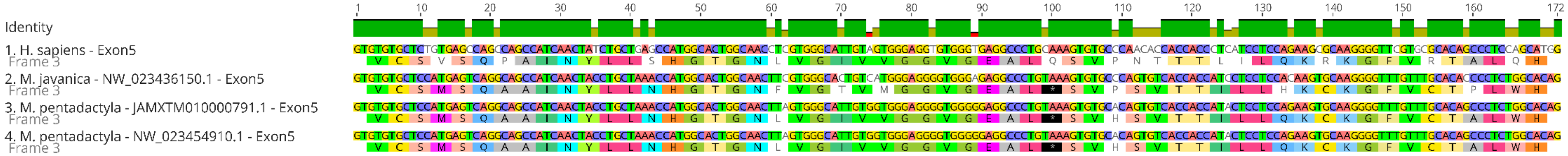

Exon 6

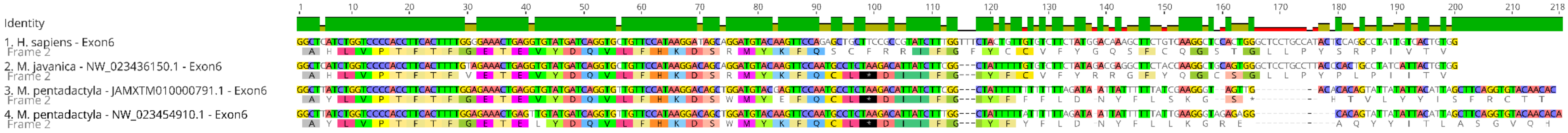

Exon 7

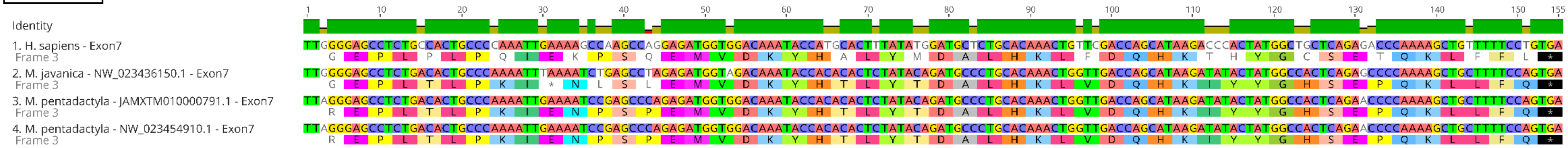

Multiple sequence alignment of each exon from AWAT1 in both *Homo sapiens* (reference), *Manis javanica* (NW_023436150.1) and *Manis pentadactyla* (NW_023454910.1 and JAMXTM010000791.1). Matches in nucleotides and aminoacids are highlighted with a specific color. Gaps (-) present in any of the *Manis* species and not in Human represent deletions, and on the other hand gaps present in Human but not in *Manis* represent insertions. Premature stop codons are represented with (*).

On the top of each exonic analysis there is a graph (denoted in different shades of green) related with the degree of similarity in each nucleotidic position in the exon.
