# Supplementary material for: Decay of Skin-Specific Gene Modules in Pangolins
Source: J Mol Evol. 2023 May 30;91(4):458–70. doi: 10.1007/s00239-023-10118-z (PMC10277264; doi:10.1007/s00239-023-10118-z)
Supplement: Supplementary file 6 — Supplementary file6 (PDF 258 KB) [file 239_2023_10118_MOESM6_ESM.pdf]

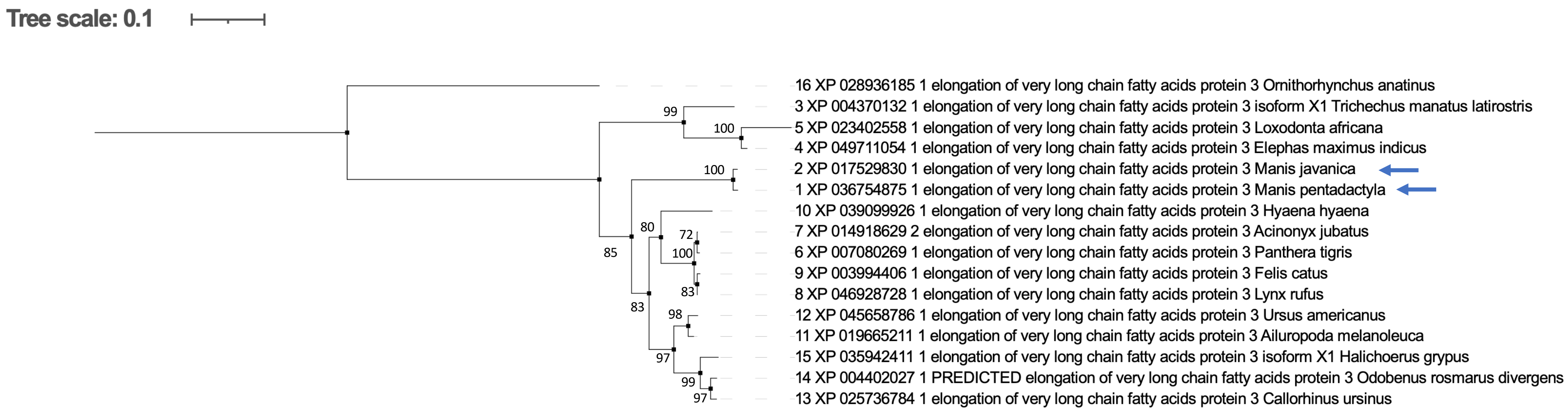

Online Resource 6. Neighbor-joining phylogenetic tree (amino acid sequence alignment produced with Mafft (defaults), JTT substitution model; numbers at nodes represent percentage of 1000 bootstraps).
